# Barriers and facilitators to implementing measurement-based care for depression in Shanghai, China: a situational analysis

**DOI:** 10.1186/s12888-021-03442-5

**Published:** 2021-09-01

**Authors:** Jill K. Murphy, Erin E. Michalak, Jing Liu, Heather Colquhoun, Hannah Burton, Xiaorui Yang, Tao Yang, Xing Wang, Yue Fei, Yanling He, Zuowei Wang, Yifeng Xu, Ping Zhang, Yousong Su, Jia Huang, Leping Huang, Lu Yang, Xiao Lin, Yiru Fang, Tianli Liu, Raymond W. Lam, Jun Chen

**Affiliations:** 1 Department of Psychiatry, University of British Columbia, Vancouver, British Columbia Canada; 2 Department of Occupational Science and Occupational Therapy, University of Toronto, Toronto, Ontario Canada; 3 Shanghai Mental Health Center, Shanghai Jiao Tong University School of Medicine, Shanghai, China; 4 Hongkou District Mental Health Center, Shanghai, China; 5 Shanghai CDC for Mental Health, Division of Training and Health Education, Shanghai, China; 6 Fengxian District Mental Health Center, Shanghai, China; 7 Peking University, Institute of Population Research, Beijing, China

**Keywords:** Measurement-based care, Digital health, Depression, Global mental health, Implementation, Situational analysis

## Abstract

**Background:**

Measurement-based care (MBC) is an evidence-based practice for depression, but its use by clinicians remains low. Enhanced MBC (eMBC), which uses digital technologies, can help to facilitate the use of MBC by clinicians and patients. Understanding factors that act as barriers and drivers to the implementation of MBC and eMBC is important to support the design of implementation strategies, promoting uptake by clinicians and patients.

**Objective:**

This situational analysis identifies barriers and facilitators to the implementation of standard and eMBC at mental health centers in Shanghai, China.

**Methods:**

We used mixed methods to develop a comprehensive understanding of the factors influencing MBC and eMBC implementation in Shanghai. This study took place across three mental health centers in Shanghai. We used situational analysis tools to collect contextual information about the three centers, conducted surveys with *n* = 116 clinicians and *n* = 301 patients, conducted semi-structured interviews with n = 30 clinicians and six focus groups with a total of *n* = 19 patients. Surveys were analysed using descriptive statistics, and semi-structured interviews and focus groups were analysed using framework analysis.

**Results:**

Several potential barriers and facilitators to MBC and eMBC implementation were identified. Infrastructure, cost, attitudes and beliefs, and perceptions about feasibility and efficacy emerged as both challenges and drivers to MBC and eMBC implementation in Shanghai.

**Conclusions:**

The results of this study will directly inform the design of an implementation strategy for MBC and eMBC in Shanghai, that will be tested via a randomized controlled trial. This study contributes to the emerging body of literature on MBC implementation and, to the best of our knowledge, is the first such study to take place in Asia. This study identifies several factors that are relevant to the equitable delivery of MBC, recognizing the need to explicitly address equity concerns in global mental health implementation research.

## Introduction and background

Measurement-based care (MBC) represents a valuable, evidence-based practice (EBP) in the treatment of major depressive disorder (MDD). It is well demonstrated that the routine use of simple, validated depression outcome scales to guide clinical decision-making can improve patient clinical outcomes [1] and treatment adherence [2]. However, despite policy-level recommendations for broad implementation of MBC [2], only a minority of clinicians avail of it [3, 4]. Barriers impeding the application of standard MBC in clinical practice include lack of clinician knowledge about appropriate scales to use, access to simple decision algorithms based on MBC, and time to administer scales. Health systems are also often not equipped to effectively track depression outcomes over time [3, 5].

Digital health technologies offer one compelling route to narrowing the MBC implementation gap [6]. In the Canadian context, we have promoted the concept of enhanced MBC (eMBC), in which digital health tools are used to facilitate uptake of MBC in both clinicians and patients.

The utility of eMBC is demonstrated by increasing evidence in the North American context [7]. While the impact of standard MBC has been illustrated in China [8], applications of eMBC in this context remain to be fully explored. This represents a key opportunity; over 54 million people experience depression in China [9], where MDD is the second-most commonly diagnosed disorder (after heart disease) and is estimated to cost 52 billion yuan (US$8.35 billion) annually [10]. China’s mental health care resources have seen rapid development over the past three decades, but greater capacity is needed [11]. Most of the mental health resources, including psychiatrists and psychiatric inpatient beds, are concentrated within the larger cities, leaving the vast suburban and rural regions with few services for mental health treatment, and most psychiatric services in China still primarily serve patients with psychotic disorders. Yet, there are also indicators to suggest that China is primed to scale up in the area of EBP. Many general hospitals have begun offering mental health services for patients with common mental disorders such as MDD. Chinese guidelines for the treatment of MDD have been recently revised and the National Mental Health Work Plan 2015–2020 specifies targets of improving the ability of healthcare facilities to identify depression and increasing the treatment rate by 50% [12]. Shanghai, China’s most populous city, has relatively strong capacity for mental health service delivery, with care delivered at three levels ranging from the Shanghai Mental Health Center (SMHC), a tertiary hospital, to district mental health centers and neighbourhood clinics. Initial steps to implement standard MBC have been undertaken in Shanghai and conditions, such as the use of electronic medical records (EMR) in many mental health centers, indicate considerable promise for adoption of eMBC across Shanghai’s mental health system. The development of an effective implementation strategy to promote the uptake and scale-up of MBC, however, requires a thorough understanding of the context of the use and delivery of depression services in Shanghai.

While evidence of the effectiveness of mental health interventions globally has increased in the last decade, there remains a gap in uptake and scale-up of EBP. To address this gap, implementation science is increasingly employed in the field of global mental health (GMH). Situational analyses are a critical first step in implementation science for GMH, as they provide a thorough and nuanced understanding of the complex context of delivery [13, 14], allowing for the development of targeted implementation strategies. Health equity considerations lie at the heart of GMH [15]. Approaches to GMH implementation research that include equity as a key consideration [16] are required to fully address current inequities in mental health service delivery and access. However, our recent scoping review of methodological approaches to situational analysis in GMH identified that minimal attention is given to health equity considerations [14]. Situational analyses provide an opportunity to identify, and subsequently to ameliorate, equity gaps to ensure implementation strategies can be tailored to promote equitable access to care.

The Enhanced Measurement-Based Care Effectiveness for Depression: A Canada-China Implementation Project (EMBED) was designed to develop a novel EBP implementation strategy by adapting, implementing and evaluating eMBC in diverse community mental health centers in Shanghai, China, modeled on programs implemented in Canada. EMBED addresses 4 broad aims: 1) identify contextual enablers & barriers to MBC implementation; 2) explore physician- and patient-level factors as mediators for an EBP implementation; 3) provide clinical and health economic outcomes to establish effectiveness of eMBC; and 4) build knowledge and capacity for scale up of eMBC in China and beyond. This formative study address the first two objectives by providing an in-depth situational analysis of the context of implementation of MBC and eMBC in Shanghai, China. The results of the situational analysis will inform the EMBED implementation strategy, which will be tested in a randomized controlled trial (RCT).

## Methods

This Situational Analysis used mixed methods to develop a comprehensive understanding of the implementation context for MBC and eMBC in Shanghai mental health centers (MHCs). Mixed methods can help to mitigate the challenges of data collection including low availability of health system data [14], provide a deeper contextual understanding and increase validity of results [17]. Specifically, three methods of data collection were used to triangulate data and ensure a comprehensive understanding of the implementation context: situational analysis tools, quantitative methods and qualitative methods.

Primary data collection took place in Shanghai, China between February and August 2019 at three mental health centers. Data were collected by members of the Shanghai research team with support from Canadian team members.

### Situational analysis tools

We developed a situational analysis (SA) tool, administered to collect data at each of the three study health centers. Shanghai Mental Health Center (SMHC) is a large, tertiary mental health and teaching hospital and is a WHO Collaborating Center. Hongkou Mental Health Center (HKMC) is a community mental health center located in the Hongkou district of central Shanghai, while Fengxian Mental Health Center (FXMC) is a community mental health center located in the suburban district of Fengxian. The SA tool was used to collect current information on coverage and service provision, workflow and readiness for MBC implementation and to capture variation between study sites, based on reports from MHC staff and internal reporting systems. The SA tool was completed by members of the Chinese study team between November 2018 and April 2019.

### Quantitative methods

Online surveys were conducted with two groups of participants, clinicians and patients, at the three MHCs between February and July 2019. To be eligible, clinician participants were required to be employed at one of the three centers and to provide informed consent. The clinician surveys included components assessing knowledge and beliefs about MBC, current practice related to use of MBC, perceived feasibility of using MBC, acceptability of eMBC, demographics and work-flow. The questions related to acceptability of eMBC were preceded by a short vignette that described the use of eMBC in a clinical setting.

Patient participants were required to have previously received a diagnosis of depression, to be between the ages of 18–65 years, to have sufficient literacy to complete the survey, and to provide informed consent. The patient survey included components assessing the use of Internet and mobile technology, the use of the Internet for health information and support, MBC and eMBC acceptability, and demographic information. The sections on MBC and eMBC acceptability were preceded by brief vignettes describing the use of MBC and eMBC in a clinical setting. Clinician surveys were distributed via private WeChat groups at each health center, with a quick response (QR) code to scan to access the survey. Patients were informed of the online survey by their clinician. Participants were asked to provide informed consent and were unable to proceed with the survey without doing so.

### Qualitative methods

Qualitative data collection occurred via semi-structured interviews with clinicians (*n* = 30) to understand their current knowledge and practice related to MBC and barriers and drivers to the use of MBC and eMBC. Six focus groups were conducted with patients at the three mental health centers, with two at each center. Focus groups were stratified by age (under and over 45 years), as age was considered a potential factor affecting uptake and use of digital technologies. The interviews and focus groups were conducted by members of the Shanghai research team, digitally recorded and transcribed in Mandarin by the Shanghai team. De-identified transcripts were then transferred to the Canadian team via a secure data transfer system, after which they were transcribed into English by two bilingual research assistants.

### Quantitative analyses

The results of the surveys were analysed by SPSS using summary statistics disaggregated by MHC location and age and gender of clinicians and patients.

### Qualitative analyses

Interviews and focus groups were analysed (by JM and EM) using a framework analysis approach, which is commonly used in health systems research to identify barriers and drivers [18], using NVivo 11 software [19]. Although data collection and analysis often occur concurrently in qualitative traditions, this was not fully feasible in this project due to the time lag for interview/focus group translations. JM and EM independently coded 6 clinician interviews and one patient focus group before meeting to develop the initial coding framework. A series of meetings then occurred to iteratively discuss and monitor coding consistency and to address the analytic validity of identified themes. A written description of the derived themes were then sent to the Shanghai team in order for them to comment on interpretations.

## Results

### SA tools

#### Coverage, uptake and service provision

Shanghai’s community MHCs operate on a catchment basis, with HKMC serving a population of 0.8 million and FXMC serving an area of 1.2 million. SMHC offers mental health services to the population of Shanghai, covering 24 million people. In 2017, HKMC saw 1066 patients per month, 410 of whom were inpatients, FXMC saw 2376 (22 inpatients) and SMHC saw 858,900 (6681 inpatients). Regarding patients accessing care for depression, in 2017 3824 accessed care at HKMC, 797 at FXMC and 40,527 at SMHC. All MHCs provide inpatient and outpatient care, and a variety of individual and group psychosocial therapies. HKMC also provides access to peer support programs. Both HKMC and FXMC have a partnership with a program (Yangguang Xinyuan) run by the Shanghai Federation of Disabled Persons, which provides community programs for people in recovery from severe depression. SMHC offers more extensive programs for inpatients (e.g. art and music therapy, occupational therapy, Morita treatment) and outpatients (cognitive behavioural therapy, social skills development, self-management and behavioural activation, music and art therapy, etc.)

100% of psychotropic medicines can be purchased at MHC pharmacies, with cost varying substantially by type of medication. HKMC and FXMC report that the average cost of medications per patient is approximately 1200 RMB (172 USD) for 6 months, while SMHC reports the average cost for the same time period as 3600 RMB (516 USD). FXMC and HKMC patients may access up to 5000 RMB per year via support by the Federation of Disabled Persons and the “Free Drug Delivery to the Countryside” program. All MHCs report that almost all patients have some insurance coverage, which may vary by type and amount of coverage for prescription medications. Therefore, while psychotropic medications are widely available, the type of medication prescribed may be influenced by cost and ability to pay.

Management and supervision at MHCs takes place on several levels. Internal departmental supervision includes intradepartmental reporting and follow-up on any areas requiring improvement. Supervision also takes place between departments, with interdepartmental recommendations to address challenges or problems. Finally, a number of higher authorities, including the Health Supervision Office, the District Health Planning Committee, the Medical Records Quality Control Committee, the Medical Insurance Office and the Price Control and Management Office conduct both regular supervision and random inspections.

### Workflow

Understanding workflow across the mental health centers is useful for understanding how MBC might be administered in practice and for planning for MBC protocols. The processes of registration, intake, assessment and referral are fairly standard across all three health centers. Because SMHC is the top tier mental health hospital in Shanghai, patients with complex treatment requirements or whose families prefer more specialized care might be referred there by HKMC and FXMC. Patients generally have a choice of physicians based on physician profiles (posted in the outpatient hallway, on the MHC’s website and on the official WeChat channel of the MHC), recommendations by nurses or other intake staff, or recommendations by friends or family members. Diverse staff are involved in initial intake, appointments and follow up visits, including intake nurses, psychiatrists, psychometricians, and pharmacists. There is some variation in booking processes, with SMHC offering online and app-based options (e.g. WeChat, Alipay, guahao.com), while HKMC and FXMC have a phone or in-person booking system with appointment records stored on health center computers. Patients visiting all MHCs have the option to pre-book or drop in. Patients can also choose their preferred clinician both initially and for follow-up appointments. In the case of HKMC, patients will only see the same clinician during follow-up by request, meaning that they often see a different clinician during initial and follow-up appointments. At FXMC, patients will generally see the same clinician at each visit unless they request a different physician. Wait times for appointments range between half an hour and 2 hours across all centers, and are unaffected by ability to pay. While SMHC and HKMC use EMR, FXMC uses EMR for inpatients but paper records for outpatients.

### Readiness for MBC implementation

At SMHC and HKMC electronic medical records are used and are shared within each hospital. FXMC uses paper patient records for outpatients and electronic prescriptions and uses electronic records for inpatients. Patients at all MHCs will receive a paper copy of their patient record following their appointment or discharge from an inpatient ward. Standards for keeping patient records are in place across Shanghai and are outlined in the Shanghai Mental Health Clinical Quality Control Manual. SMHC indicates that they have a dedicated staff person providing oversight for quality of patient records. Computers are available at all centers to all clinicians and Internet access is available across all MHC’s but may be inconsistent at FXMC, the suburban center, which uses a LAN connection that can be unreliable. Standardized measures are available both on paper and online at each MHC and are currently administered only by psychometricians. Commonly used measures include the Zung Self-Rating Depression and Anxiety Scales [20], Symptom Checklist (SCL)-90 [21], the Minnesota Multiphasic Personality Inventory (MMPI) [22], Eysenck’s personality questionnair(EPQ), and the Hamilton Depression Rating Scale (HAMD) [23].

#### Survey and qualitative data

Below, survey and qualitative data results are presented for the clinician and patient groups. Emergent themes from the qualitative data are supported by direct quotes.

### Mixed methods results: clinicians

A total of *N* = 116 clinicians completed the online survey (SMHC *n* = 78, HKMC *n* = 23, FXMC *n* = 15). Demographic information, qualifications and workload are described in Table 1:

**Table 1:** Clinician demographics, qualifications and work load

Survey Question	Response	SMHC: n (%)	HKMC: n (%)	FXMC: n (%)	Total: n (%)
**Age Range**	18–29	0	0	4 (26.7)	4 (3.4)
	30–39	34 (43.6)	11 (47.8)	9 (60.0)	54 (46.6)
	40–49	31 (39.7)	12 (52.2)	1 (6.7)	44 (37.9)
	50–59	13 (16.7)	0	1 (6.7)	14 (12.1)
	**Total**	**78 (100)**	**23 (100)**	**15 (100)**	**116 (100)**
**Gender**	Female	51 (65.4)	19 (82.6)	10 (66.7)	80 (69.0)
	Male	27 (34.6)	4 (17.4)	5 (33.3)	36 (31.0)
	**Total**	**78 (100)**	**23 (100)**	**15 (100)**	**116 (100)**
**Highest degree**	Junior college	0	2 (8.7)	1 (6.7)	3 (2.6)
	Bachelor’s	14 (17.9)	11 (47.8)	13 (86.7)	38 (32.8)
	Master’s	36 (46.2)	8 (34.8)	1 (6.7)	45 (38.8)
	Doctorate	28 (35.6)	2 (8.7)	0	30 (25.9)
	**Total**	**78 (100)**	**23 (100)**	15 (100)	**116 (100)**
**Professional group**	Psychiatrist	78 (100)	23 (100)	15 (100)	116 (100)
	General practitioner	0	0	0	0
	Neurologist	0	0	0	0
	Psychologist	0	0	0	0
	Social worker	0	0	0	0
	Nurse	0	0	0	0
	Nurses assistant	0	0	0	0
	Other	0	0	0	0
	**Total**	**78 (100)**	**23 (100)**	**15 (100)**	**116 (100)**
**Primary work**	Hospital OPC^a^	28 (35.9)	4 (17.4)	0	32 (27.6)
	Hospital IPU^b^	9 (11.5)	11 (47.8)	15 (100)	35 (30.2)
	Hospital OPC and IPU	35 (44.9)	7 (30.4)	0	42 (36.2)
	Chronic disease centre	1 (1.3)	1 (4.3)	0	2 (1.7)
	Other	5 (6.4)	0	0	5 (4.3)
	**Total**	**78 (100)**	**23 (100)**	**15 (100)**	**116 (100)**
**Years in job**	< 1 year	0	0	1 (6.7)	1 (0.9)
	1–5	1 (1.3)	3 (13.0)	3 (20.0)	7 (6.0)
	6–10	15 (19.2)	4 (17.4)	6 (40.0)	25 (21.6)
	11–15	18 (23.1)	6 (26.1)	4 (26.7)	28 (24.1)
	16–20	22 (28.2)	6 (26.1)	0	28 (24.1)
	> 20	22 (28.2)	4 (17.4)	1 (6.7)	27 (23.3)
	**Total**	**78 (100)**	**23 (100)**	**15 (100)**	**116 (100)**
**Hours/ week in patient care**	< 8	5 (6.4)	2 (8.7)	3 (20.0)	10 (8.6)
	8–20	18 (23.1)	4 (17.4)	5 (33.3)	27 (23.3)
	20–30	10 (12.8)	4 (17.4)	4 (26.7)	18 (15.5)
	30–40	24 (30.8)	8 (34.8)	3 (20.0)	35 (30.2)
	> 40	21 (26.9)	5 (21.7)	0	26 (22.4)
	**Total**	**78 (100**)	**23 (100)**	**15 (100)**	**116 (100)**
**Average # of patients/ week**	20–50	14 (36.2)	14 (60.9)	14 (93.3)	42 (36.2)
	50–80	17 (18.1)	3 (13.0)	1 (6.7)	21 (18.1)
	80–110	22 (20.7)	2 (8.7)	0	24 (20.7)
	110–140	10 (10.3)	2 (8.7)	0	12 (10.3)
	140–160	4 (3.4)	0	0	4 (3.4)
	160–200	5 (4.3)	0	0	5 (4.3)
	> 200	6 (6.9)	2 (8.7)	0	8 (6.9)
	**Total**	**78 (100)**	**23 (100)**	**15 (100)**	**116 (100)**
**Primary specialization**	Mood disorders	15 (19.8)	7 (30.4)	1 (6.7)	23 (19.8)
	Clinical psychology	10 (10.3)	1 (4.3)	1 (6.7)	12 (10.3)
	Geriatric psychiatry	4 (5.2)	0	2 (13.3)	6 (5.2)
	Child and adolescent psychiatry	7 (6.9)	1 (4.3)	0	8 (6.9)
	General psychiatry	40 (55.2)	13 (56.5)	11 (73.3)	64 (55.2)
	Substance use disorders	2 (2.6)	1 (4.3)	0	3 (2.6)
	**Total**	**78 (100)**	**23 (100)**	**15 (100)**	**116 (100)**

^a^Outpatient clinic; ^b^Inpatient unit

Though all survey participants indicated that they are psychiatrists, their highest degree of qualification varied. This is likely related to the change in training requirements over time, as prior to 2000 it was possible to work as a psychiatrist without a Bachelor’s degree (subsequently, training requirements have become more stringent). Primary context of work varied across MHCs. At SMHC, 35.9% of surveyed clinicians work in an outpatient center (OPC), 11.5% in an inpatient unit (IPU) and 44.9% in both, at HKMC, 17.4% work in an OPC, 47.8% in an IPU and 30.4% in both, while at FXMC 100% of surveyed clinicians work in an IPU. Surveyed clinicians were fairly experienced, with 23.3% having spent between 6 and 10 years, 24.1% 11–15 years, 24.1% 16–20 years, and 23.3% over 20 years in practice. FXMC’s clinicians were somewhat newer to their jobs, with 40% indicating they has been working for 6–10 years, while at SMHC 28.2% have been working for over 20 years. Shanghai clinicians spend a substantial amount of time in patient care, with 30.2% spending 30–40 h per week and 22.4% spending over 40 h per week, though clinicians at FXMC reported less time with patients compared with SMHC and HKMC. Numbers of patients per week was also lower at FXMC, as shown in Table 1. Clinicians at SMHC saw the highest volumes of patients per week. A majority of clinicians surveyed (55.2%) specialized in general psychiatry, with 73.3% at FXMC selecting this as their specialization. The other most common categories of specialization are mood disorders (19.8%) and clinical psychology (10.3%).

### Current practice related to MBC

Through surveys and interviews we assessed clinicians’ current practice related to MBC, including how they currently assess changes in a patient’s condition, medication side effects and functional ability, and their current knowledge about MBC. Surveyed clinicians were asked to rate their level of agreement on questions related to their current practice in terms of MBC use, as described in Table 2:

**Table 2:** Clinician current practice of MBC

Survey Question	Response	Location			Age		Gender		
		SMHC: n (%)	HKMC: n (%)	FXMC: n (%)	18–39 years	40–59 years	F	M	Total: n (%)
**MBC is important and useful to me in addition to my own clinical judgement**	Strongly disagree	0	0	0	0	0	0	0	0
	Disagree	0	0	0	0	0	0	0	0
	Somewhat disagree	7 (9.0)	1 (4.3)	1 (6.7)	5 (8.6)	4 (6.9)	7 (8.8)	2 (5.6)	9 (7.8)
	Somewhat agree	21 (26.9)	8 (34.8)	1 (6.7)	13 (22.4)	17 (29.3)	16 (20.0)	14 (38.9)	30 (25.9)
	Agree	42 (53.8)	11 (47.8)	13 (86.7)	33 (56.9)	33 (56.9)	49 (61.3)	17 (47.2)	66 (56.9)
	Strongly agree	8 (10.3)	3 (13.0)	0	7 (12.1)	4 (6.9)	8 (10.0)	3 (8.3)	11 (9.5)
	**Total**	**78 (100)**	**23 (100)**	**15 (100)**	**58 (100)**	**58 (100)**	**80 (100)**	**36 (100)**	**116 (100)**
**I am trained in the use of MBC and how to use scores for clinical decisions.**	Strongly disagree	3 (3.8)	0	0	1 (1.7)	2 (3.4)	1 (1.3)	2 (5.6)	3 (2.6)
	Disagree	21 (26.9)	5 (21.7)	2 (13.3)	18 (31.0)	10 (17.2)	21 (26.3)	7 (19.4)	28 (24.1)
	Somewhat disagree	11 (14.1)	1 (4.3)	0	5 (8.6)	7 (12.1)	8 (10.0)	4 (11.1)	12 (10.3)
	Somewhat agree	13 (16.7)	4 (17.4)	3 (20.0)	9 (15.5)	11 (19.0)	16 (20.0)	4 (11.1)	20 (17.2)
	Agree	24 (30.8)	9 (39.1)	10 (66.7)	22 (37.9)	21 (36.2)	29 (36.3)	14 (38.9)	43 (37.1)
	Strongly agree	6 (7.7)	4 (17.4)	0	3 (5.2)	7 (12.1)	5 (6.3)	5 (13.9)	10 (8.6)
	**Total**	**78 (100)**	**23 (100)**	**15 (100)**	**58 (100)**	**58 (100)**	**80 (100)**	**36 (100)**	**116 (100)**
**I have sufficient knowledge to be able to interpret MBC scores.**	Strongly disagree	0	0	0	0	0	0	0	0
	Disagree	4 (5.1)	2 (8.7)	0	4 (6.9)	2 (3.4)	4 (5.0)	2 (5.6)	6 (5.2)
	Somewhat disagree	6 (7.7)	1 (4.3)	2 (13.3)	4 (6.9)	5 (8.6)	5 (6.3)	4 (11.1)	9 (7.8)
	Somewhat agree	21 (26.9)	5 (21.7)	4 (26.7)	16 (27.6)	14 (24.1)	24 (30.0)	6 (16.7)	30 (25.9)
	Agree	41 (52.6)	14 (60.9)	9 (60.0)	32 (55.2)	32 (55.2)	45 (56.3)	19 (52.8)	64 (55.2)
	Strongly agree	6 (7.7)	1 (4.3)	0	2 (3.4)	5 (8.6)	2 (2.5)	5 (13.9)	7 (6.0)
	**Total**	**78 (100)**	**23 (100)**	**15 (100)**	**58 (100)**	**58 (100)**	**80 (100)**	**36 (100)**	**116 (100)**
**I use MBC at each visit to monitor the course of treatment for patients.**	Strongly disagree	5 (6.4)	1 (4.3)	0	2 (3.4)	4 (6.9)	3 (3.8)	3 (8.3)	6 (5.2)
	Disagree	18 (23.1)	5 (21.7)	0	14 (24.1)	9 (15.5)	18 (22.5)	5 (13.9)	23 (19.8)
	Somewhat disagree	21 (26.9)	3 (13.0)	0	9 (15.5)	15 (25.9)	15 (18.8)	9 (25.0)	24 (20.7)
	Somewhat agree	21 (26.9)	10 (43.5)	4 (26.7)	18 (31.0)	17 (29.3)	26 (32.5)	9 (25.0)	35 (30.2)
	Agree	11 (14.1)	4 (17.4)	11 (73.3)	14 (24.1)	12 (20.7)	17 (21.3)	9 (25.0)	26 (22.4)
	Strongly agree	2 (2.6)	0	0	1 (1.7)	11 (19.0)	1 (1.2)	1 (2.8)	2 (1.7)
	**Total**	**78 (100)**	**23 (100)**	**15 (100)**	**58 (100)**	**58 (100)**	**80 (100)**	**36 (100)**	**116 (100)**
**I discuss the MBC scores with my patients.**	Strongly disagree	1 (1.3)	1 (4.3)	0	1 (1.7)	1 (1.7)	2 (2.5)	0	2 (1.7)
	Disagree	6 (7.7)	1 (4.3)	0	6 (10.3)	1 (1.7)	6 (7.5)	1 (2.8)	7 (6.0)
	Somewhat disagree	10 (12.8)	5 (21.7)	0	10 (17.2)	5 (8.6)	11 (13.8)	4 (11.1)	15 (12.9)
	Somewhat agree	27 (34.6)	12 (52.2)	3 (20.0)	15 (25.9)	27 (46.6)	29 (36.3)	13 (36.1)	42 (36.2)
	Agree	30 (38.5)	4 (17.4)	12 (80.0)	24 (41.4)	22 (37.9)	30 (37.5)	16 (44.4)	46 (39.7)
	Strongly agree	4 (5.1)	0	0	2 (3.4)	2 (3.4)	2 (2.5)	2 (5.6)	4 (3.4)
	**Total**	**78 (100)**	**23 (100)**	**15 (100)**	**58 (100)**	**58 (100)**	**80 (100)**	**36 (100)**	**116 (100)**

Survey responses on current practice with MBC varied, with clinicians across mental health centers indicating a high level of agreement regarding the importance of MBC, their knowledge about interpreting MBC scores, and the importance of discussing MBC results with patients, as shown in Table 2. Reported levels of training in the use of MBC varied considerably, with 24.1% of total clinicians surveyed indicating they disagree that they are trained in the use of MBC for clinical decision making, while 37.1% indicated that they agree to being sufficiently trained. While responses from SMHC and HKMC were similar, a higher proportion of FXMC clinicians indicated they were sufficiently trained, with 66.7% agreeing.

Survey results were variable in terms of their use of MBC at each visit. 19.8% of clinicians disagree and 20.7% somewhat disagreed that they used MBC at each visit, while 30.2% somewhat agreed and 22.4% agreed. Notably 73.3% of FXMC clinicians, compared with 14.1% at SMHC and 14.5% at HKMC agreed that they use MBC at each visit. This is consistent with the results of qualitative interviews, where descriptions of use of standardized assessment scales as part of routine clinical care varied highly by center. At SMHC measures were typically only applied during initial consults; administration of depression scales over time to monitor response to treatment or longitudinally track outcomes was uncommon.

At HKMC and FXMC descriptions of use of outcome measures in the initial appointment or subsequently to assess changes in a patients’ depression severity, treatment side effects or functioning varied. While some described using measures, a majority stated that they are used occasionally or not at all. For example, a clinician from FXMC stated that: *“for some patients, we’ll ask them to do some measures, but very seldom”.* (FX01).

Others described using it occasionally on inpatient wards but never in outpatient clinics. Some clinicians described measures being used more frequently during initial visits but not during subsequent appointments:

*“We also use the standardized outcome measures often. More frequently we use them in the initial assessments or when there are changes of medical conditions. Generally speaking, we do not use them for every regular visit. Especially when the medical condition is quite stable, measures are seldom used”* (HK05)

Patient report was the most commonly referred to approach to assessing changes in depression severity as shown in the following quote:

*“According to my clinical experience, then I will listen to his main complaints, includ(ing) observing some of his external performances, such as expression, tone of voice”. (SH03)*

Family report of a patient’s condition was also frequently described as a means of assessing changes in depression severity, as described by a clinician from Fengxian:

*“When patients come to the visits, I’d ask about their status at home, including sleep, eating, work, and relationships with their family, as well as mood changes. After that, I’ll ask their family to add some medical conditions.” (FX06)*

A clinician from Hongkou stated that they assess changes in a patient’s condition “from the information provided by people surround[ing] that patient” (*HK10*) while a SMHC clinician stated that “[c]ollected information is mainly from their family” (*SH08*).

Some clinicians did describe using measures, though they were in the minority. A clinician from Hongkou MHC described: *“The patient’s condition changes can be assess(ed) through some measures, such as the Hamilton Depression Scale, etc., we can see whether his condition has changed, whether it is improved or worsened”. (HK08).*

When measures were utilised, they were on occasion described as being *“auxiliary”* (SH07) or *“correlative”* (*SH01*).

A majority of clinicians interviewed also described using patient report or family feedback to assess the impact of side effects:

*“Mainly based on the information provided by patients or their families after they take the medications, as well as patients’ own feelings/experience”. (SH02)*

Several clinicians described the use of measures to assess side effects, but did not describe their use as part of their routine clinical practice:

*“First, we’ll focus on their main complaints. When they come, they’ll tell you some current symptoms. If they do not mention these actively, then we’ll ask them whether they have some physical discomforts or not, and ask them to think about whether these are related to the meds. If necessary, we’ll also do some measures.” (FX09)*

One respondent from Fengxian described using measures to assess side effects in approximately one of 10 patients.

In terms of assessing improvements in patients’ functional ability, the majority of clinicians again described patient and family report as the most common means of assessment:

*“We usually ask about patients’ (abilities) such as work ability, as well as their general symptoms, but the most important thing is to ask their family, because some depression patients may have a different self-assessment of their abilities. Hence, we mainly ask their families, about their general daily performance at home, as well as work ability, etc.” (FX06)*

Three clinicians described using measures to assess functional ability, but using functional measures was not common practice, for example:

*“ [We can] identify the changes by measurement assessment. Such as the functional ability measure that we have now.” (HK03) and “Clinicians will assess the patient from some social function measures as well” (SH01)*

### Knowledge and beliefs about MBC

Clinicians were also asked to respond to survey questions about their knowledge and beliefs about MBC, as described in Table 3.

**Table 3:** Clinician Knowledge and Beliefs about MBC

Survey Question	Response	Location			Age		Gender		Total: n (%)
		SMHC: n (%)	HKMC: n (%)	FXMC: n (%)	18–39 years	40–59 years	F	M	
**Standardized questionnaires and scales are valid (they measure what they are supposed to measure) and reliable (they give the same score with repeated use under the same conditions) assessments of symptom severity.**	Strongly disagree	0	0	0	0	0	0	0	0
	Disagree	0	0	0	0	0	0	0	0
	Somewhat disagree	3 (3.8)	0	1 (6.7)	3 (5.2)	1 (1.7)	2 (2.5)	2 (5.6)	4 (3.4)
	Somewhat agree	7 (9.0)	2 (8.7)	0	6 (10.3)	3 (5.2)	6 (7.5)	3 (8.3)	9 (7.8)
	Agree	56 (71.8)	15 (65.2)	12 (80.0)	40 (69.0)	43 (74.1)	60 (75)	23 (63.9	83 (71.6)
	Strongly agree	12 (15.4)	6 (26.1)	2 (13.3)	9 (15.5)	11 (19.0)	12 (15)	8 (22.2)	20 (17.2)
	**Total**	**78 (100)**	**23 (100)**	**15 (100)**	**58 (100)**	**58 (100)**	**80 (100)**	**36 (100)**	**116 (100)**
**MBC can improve patient outcomes.**	Strongly disagree	0	0	0	0	0	0	0	0
	Disagree	5 (6.4)	1 (4.3)	0	3 (5.2)	3 (5.2)	3 (3.8)	3 (8.3)	6 (5.2)
	Somewhat disagree	7 (9.0)	1 (4.3)	1 (6.7)	6 (10.3)	3 (5.2)	6 (7.5)	3 (8.3)	9 (7.8)
	Somewhat agree	17 (21.8)	5 (21.7)	2 (13.3)	9 (15.5)	15 (25.9)	18 (22.5)	6 (16.7)	24 (20.7)
	Agree	42 (53.8)	15 (65.2)	11 (73.3)	36 (62.1)	31 (53.4)	46 (57.5)	21 (58.3	67 (57.8)
	Strongly agree	7 (9.0)	3 (13.0)	1 (6.7)	4 (6.9)	6 (10.3)	7 (8.8)	3 (8.3)	10 (8.6)
	**Total**	**78 (100)**	**23 (100)**	**15 (100)**	**58 (100)**	**58 (100)**	**80 (100)**	**36 (100)**	**116 (100)**
**MBC is helpful for making treatment decisions.**	Strongly disagree	0	0	0	0	0	0	0	0
	Disagree	1 (1.3)	0	0	0	1 (1.7)	0	1 (2.8)	1 (0.9)
	Somewhat disagree	2 (2.6)	0	1 (6.7)	2 (3.4)	1 (1.7)	2 (2.5)	1 (2.8)	3 (2.6)
	Somewhat agree	15 (19.2)	5 (21.7)	2 (13.3)	10 (17.2)	12 (20.7)	13 (18.8)	7 (19.4)	22 (19.0)
	Agree	49 (62.8)	15 (65.2)	8 (53.3)	37 (63.8)	35 (60.3)	50 (62.5)	22 (61.1)	72 (62.1)
	Strongly agree	11 (14.1)	3 (13.0)	4 (26.7)	9 (15.5)	9 (15.5)	13 (16.3)	5 (13.9)	18 (15.5)
	**Total**	**78 (100)**	**23 (100)**	**15 (100)**	**58 (100)**	**58 (100)**	**80 (100)**	**36 (100)**	**116 (100)**
**MBC at each visit is helpful for monitoring treatment response.**	Strongly disagree	0	0	0	0	0	0	0	0
	Disagree	0	0	0	0	0	0	0	0
	Somewhat disagree	2 (2.6)	1 (4.3)	2 (13.3)	3 (5.2)	2 (3.4)	4 (5.0)	1 (2.8)	5 (4.3)
	Somewhat agree	14 (17.9)	3 (13.0)	0	9 (15.5)	8 (13.8)	11 (13.8)	6 (16.7)	17 (14.7)
	Agree	50 (64.1)	16 (69.6)	10 (66.7)	36 (62.1)	40 (69.0)	51 (63.7)	25 (69.4	76 (65.5)
	Strongly agree	12 (15.4)	3 (13.0)	3 (20.0)	10 (17.2)	8 (13.8)	14 (17.5)	4 (11.1)	18 (15.5)
	**Total**	**78 (100)**	**23 (100)**	**15 (100)**	**58 (100)**	**58 (100)**	**80 (100)**	**36 (100)**	**116 (100)**
**MBC enhances care provided to a given patient.**	Strongly disagree	0	0	0	0	0	0	0	0
	Disagree	2 (2.6)	0	0	1 (1.7)	2 (3.4)	2 (2.5)	1 (2.8)	3 (2.6)
	Somewhat disagree	2 (2.6)	1 (4.3)	1 (6.7)	3 (5.2)	0	3 (3.8)	0	3 (2.6)
	Somewhat agree	16 (20.5)	7 (30.4)	2 (13.3)	8 (13.8)	17 (29.3)	13 (16.3)	12 (33.3)	25 (21.6)
	Agree	49 (62.8)	11 (47.8)	10 (66.7)	38 (65.5)	32 (55.2)	52 (65.0)	18 (50.0)	70 (60.3)
	Strongly agree	9 (11.5)	4 (17.4)	2 (13.3)	8 (13.8)	7 (12.1)	10 (12.5	5 (13.9)	15 (12.9)
	**Total**	**78 (100)**	**23 (100)**	**15 (100)**	**58 (100)**	**58 (100)**	**80 (100)**	**36 (100)**	**116 (100)**
**Patients will find MBC helpful.**	Strongly disagree	0	0	0	0	0	0	0	0
	Disagree	1 (1.3)	0	1 (6.7)	1 (1.7)	1 (1.7)	2 (2.5)	0	2 (1.7)
	Somewhat disagree	4 (5.1)	4 (17.4)	0	3 (5.2)	5 (8.6)	5 (6.3)	3 (83)	8 (6.9)
	Somewhat agree	22 (28.2)	8 (34.8)	3 (20.0)	14 (24.1)	19 (32.8)	19 (23.8)	14 (38.9)	33 (28.4)
	Agree	48 (61.5)	10 (43.5)	11 (73.3)	39 (67.2)	30 (51.7)	51 (63.7)	18 (50.0)	69 (59.5)
	Strongly agree	3 (3.8)	1 (4.3)	0	1 (1.7)	3 (5.2)	3 (3.8)	1 (2.8)	4 (3.4)
	**Total**	**78 (100)**	**23 (100)**	**15 (100)**	**58 (100)**	**58 (100)**	**80 (100)**	**36 (100)**	**116 (100)**
**MBC can help educate patients about their mental health symptoms.**	Strongly disagree	0	0	0	0	0	0	0	0
	Disagree	0	0	0	0	0	0	0	0
	Somewhat disagree	2 (2.6)	1 (4.3)	0	1 (1.7)	2 (3.4)	3 (3.8)	0	3 (2.6)
	Somewhat agree	13 (16.7)	7 (30.4)	2 (13.3)	11 (19.0)	11 (19.0)	14 (17.5)	8 (22.2)	22 (19)
	Agree	50 (64.1)	12 (52.2)	12 (80.0)	39 (67.2)	35 (60.3)	52 (65.0)	22 (61.1)	74 (63.8)
	Strongly agree	13 (16.7)	3 (13.0)	1 (6.7)	7 (12.1)	10 (17.2)	11 (13.8)	6 (16.7)	17 (14.7)
	**Total**	**78 (100)**	**23 (100)**	**15 (100)**	**58 (100)**	**58 (100)**	**80 (100)**	**36 (100)**	**116 (100)**
**MBC can help patients feel more engaged in shared decision making.**	Strongly disagree	0	0	0	0	0	0	0	0
	Disagree	0	0	0	0	0	0	0	0
	Somewhat disagree	4 (5.1)	1 (4.3)	1 (6.7)	4 (6.9)	2 (3.4)	5 (6.3)	1 (2.8)	6 (5.2)
	Somewhat agree	10 (12.8)	7 (30.4)	0	5 (8.6)	12 (20.7)	10 (12.5)	7 (19.4)	17 (14.7)
	Agree	51 (65.4)	12 (52.2)	10 (66.7)	38 (65.5)	35 (60.3)	50 (62.5)	23 (63.9)	73 (62.9)
	Strongly agree	13 (16.7)	3 (13.0)	4 (26.7)	11 (19.0)	9 (15.5)	15 (18.8)	5 (13.9)	20 (17.2)
	**Total**	**78 (100)**	**23 (100)**	**15 (100)**	**58 (100)**	**58 (100)**	**80 (100)**	**36 (100)**	**116 (100)**
**MBC can help other providers care for mutual patients.**	Strongly disagree	0	0	0	0	0	0	0	0
	Disagree	1 (1.3)	0	0	0	1 (1.7)	0	1 (2.8)	1 (0.9)
	Somewhat disagree	1 (1.3)	2 (8.7)	1 (6.7)	2 (3.4)	2 (3.4)	3 (3.8)	1 (2.8)	4 (3.4)
	Somewhat agree	9 (11.5)	5 (21.7)	1 (6.7)	9 (15.5)	6 (10.3)	7 (8.8)	8 (22.2)	15 (12.9)
	Agree	55 (70.5)	10 (43.5)	9 (60.0)	34 (58.6)	40 (69.0)	53 (56.3)	21 (58.3)	74 (63.8)
	Strongly agree	12 (15.4)	6 (26.1)	4 (26.7)	13 (22.4)	9 (15.5)	17 (21.7)	17 (21.7)	22 (19.0)
	**Total**	**78 (100)**	**23 (100)**	**15 (100)**	**58 (100)**	**58 (100)**	**80 (100)**	**80 (100)**	**116 (100)**

Clinician responses to all questions related to knowledge and beliefs about MBC showed positive attitudes, with a high level of agreement regarding the validity and reliability of standardized measures, the positive impact of MBC for treatment decision-making, monitoring treatment response, patient education, patient engagement in decision-making and consistency of care across providers.

Qualitative interviews explored clinicians’ existing knowledge about MBC. Familiarity with MBC varied across health centers, with a majority at SMHC stating that they are familiar with it while at HKMC and FXMC there was more variability. Some provided definitions of it, stating that MBC is more objective and standardized, allowing for tailored treatment decisions. One clinician from Fengxian stated: *“I think if it’s measurement-based care, the treatment will be more standardized. That’s my understanding”* (*FX04*). A clinician from Hongkou gave the following description:

*“It may be relatively more standard or systematic, so it may be relatively comprehensive. It’s more standardized, not just the doctors’ feelings, right? If so (based on doctors’ feelings only), there might be some deviations. Through regular assessments, we can have a comprehensive evaluation, and it will also help us to have an overall idea of their medical conditions.” (HK01)*

Another interviewee from SMHC provided a definition that described how the comprehensive nature of MBC might benefit treatment decision-making:

*“MBC is not just about evaluating patient’s symptoms every time, but also identifying side effects, which is the most basic process…At first, we need to identify the efficacy and the side effects. Then measure the risk-benefit ratio between the two. So as to determine what kind of further treatment plan the clinician will use, like whether to increase or reduce medicine. Although MBC is somehow subjective, it is relatively more objective according to our clinical experience”. (SH03)*

Others noted that they have heard of MBC but are unfamiliar with its meaning, or that they have not heard of it at all: *“Based on measurement? Since I’m in the geriatric psychiatry department, I’m not familiar with that”* (FX08) or that they had received training on MBC but not absorbed it fully: *“There seemed to have (been) some training in our hospital, but I didn’t pay too much attention to it”. (HK07).*

Despite the survey results suggesting positive attitudes towards MBC, interviewed clinicians identified several perceived barriers to them using it in their current practice. They identified time constraints as a main barrier, as seen in the following quote from a clinician in Fengxian:

*“Now, there should be measures, measures for depression, but in outpatient clinics, sometimes time is limited, so (we are) unable to do the measures.” (FX02)*

Clinicians from HKMC and SMHC expressed the same concerns: *“But there are difficulties in the clinical practice, that is, there is not too much time for doing this” (HK07)* and *“There is no time to do it. Time is a big problem”. (SH07).*

There was also a concern that using MBC would require more time allocated to patient consultations:

*“The more information you let patients know, the more questions they will have and the more time you need to spend for explanation. Hence the first thing is that we need time”. (SH08)*

Clinicians also suggest that their patients might be reluctant to complete measures due to their own time limitations or general reluctance to do so:

*“If the questionnaire is too long, the patient seems to have no patience with it”. (HK07)*

Some clinicians also question the accuracy of measures and the ability of patients to accurately self-report, as they believe that patients might be unable to give an “objective” response:

*“...there's a huge difference between different individuals, and the scales are based on patients’ own feelings. They will fill out as what they believe their medical conditions are (which may not be the reality). Their feelings might not be very objective. Hence, the measures may not be very accurate.” (FX07)*

Other factors identified include the belief that measures are unavailable, as stated by this clinician from Hongkou: *“You mentioned the standardized outcome measure. In my knowledge and current practice, this measure has not been used, nor have I noticed it” (HK02).*

And, more specifically, concerns about lack of availability of appropriately adapted measures:

*“Different measurement tools can cause problems. If some of them are imported and translated into Chinese, they may not fit into [our] native culture”. (SH03)*

Several drivers related to clinicians’ beliefs about MBC also emerged during qualitative interviews. Improved patient empowerment, awareness about their illness and improved treatment alliance were described as potential benefits by clinicians. A clinician from Fengxian stated:

*“I think if it’s me, I’d be willing to use such an assessment, because in this way the doctors and patients will be in an equal status, and doctors will fully respect patients. Patients will have the right to know about their entire treatments. It’s about the informed consent. At least they know about their own disease better, which would benefit the following treatments.” (FX04)*

A clinician from Hongkou also stated: *“It also has the advantage of giving patients a more complete understanding of their condition, that is, letting the patient have a comprehensive understanding of himself” (HK10).*

Clinicians also noted that MBC could help to improve treatment adherence by involving patients more actively in their treatment decision-making and helping to increase their awareness about symptom changes that might go unnoticed without the systematic use of scales. *“I think the biggest benefit would be improved medical adherence, because they get involved”. (HK06).*

A Hongkou clinician described the potential for MBC to ultimately improve hope about treatment effectiveness and adherence among patients:

*“It will make it easier to identify the aspects of their improvements, and pay attention to those improvements as well instead of focusing on the unimproved ones. In this way, patients will become more faithful, which is helpful as well.” (HK01)*

A SMHC clinician similarly described the potential positive impact of seeing an objective improvement in outcomes on patient morale: *“In fact, many patients, for example, did not have a big change in the treatment of nearly two weeks, but if he pass this score, he will gain some confidence”. (SH01).*

### Feasibility and perceived barriers and facilitators of use of MBC

Clinicians were asked to rate their level of agreement on questions related to the feasibility of using MBC, as described in Table 4:

**Table 4:** Feasibility of using MBC

Survey Question	Response	Location			Age		Gender		
		SMHC: n (%)	HKMC: n (%)	FXMC: n (%)	18–39 years	40–59 years	F	M	Total: n (%)
**MBC would be easy to integrate into my daily workflow.**	Strongly disagree	1 (1.3)	0	0	1 (1.7)	0	0	1 (2.8)	1 (0.9)
	Disagree	6 (7.7)	0	0	2 (3.4)	4 (6.9)	3 (3.8)	3 (8.3)	6 (5.2)
	Somewhat disagree	10 (12.8)	2 (8.7)	1 (6.7)	8 (13.8)	5 (8.6)	9 (11.3)	4 (11.1)	13 (11.2)
	Somewhat agree	24 (30.8)	15 (65.2)	2 (13.3)	20 (34.5)	21 (36.2)	29 (36.3)	12 (33.3)	41 (35.3)
	Agree	36 (46.2)	6 (26.1)	11 (73.3)	26 (44.8)	27 (46.6)	39 (48.8)	14 (38.9)	53 (45.7)
	Strongly agree	1 (1.3)	0	1 (6.7)	1 (1.7)	1 (1.7)	0	2 (5.6)	2 (1.7)
	**Total**	**78 (100)**	**23 (100)**	**15 (100)**	**58 (100)**	**58 (100)**	**80 (100)**	**36 (100)**	**116 (100)**
***I would use MBC if my hospital provided training and resources.***	Strongly disagree	0	0	0	0	0	0	0	0
	Disagree	1 (1.3)	0	0	0	1 (1.7)	1 (1.3)	0	1 (0.9)
	Somewhat disagree	2 (2.6)	1 (4.3)	0	2 (3.4)	1 (1.7)	2 (2.5)	1 (2.8)	3 (2.6)
	Somewhat agree	17 (21.8)	5 (21.7)	1 (6.7)	9 (15.5)	14 (24.1)	15 (18.8)	8 (22.2)	23 (19.8)
	Agree	45 (57.7)	12 (52.2)	9 (60.0)	37 (63.8)	29 (50.0)	46 (57.5)	20 (55.6)	66 (56.9)
	Strongly agree	13 (16.7)	5 (21.7)	5 (33.3)	10 (17.2)	13 (22.4)	16 (20.0)	7 (19.4)	23 (19.8)
	**Total**	**78 (100)**	**23 (100)**	**15 (100)**	**58 (100)**	**58 (100)**	**80 (100)**	**36 (100)**	**116 (100)**
***I would use data collected from MBC to evaluate my own practices.***	Strongly disagree	0	0	0	0	0	0	0	0
	Disagree	0	0	0	0	0	0	0	0
	Somewhat disagree	4 (5.1)	0	0	2 (3.4)	2 (3.4)	3 (3.8)	1 (2.8)	4 (3.4)
	Somewhat agree	21 (26.9)	6 (26.1)	2 (13.3)	11 (19.0)	18 (31.0)	18 (22.5)	11 (30.6)	29 (25.0)
	Agree	44 (56.4)	14 (60.9)	11 (73.3)	40 (69.0)	29 (50.0)	50 (62.5)	19 (52.8)	69 (59.5)
	Strongly agree	9 (11.5)	3 (13.0)	2 (13.3)	5 (8.6)	9 (15.5)	9 (11.3)	5 (13.9)	14 (12.1)
	**Total**	**78 (100)**	**23 (100)**	**15 (100)**	**58 (100)**	**58 (100)**	**80 (100)**	**36 (100)**	**116 (100)**
***My patients would be willing to complete MBC at each visit.***	Strongly disagree	0	0	0	0	0	0	0	0
	Disagree	4 (5.1)	1 (4.3)	0	1 (1.7)	4 (6.9)	4 (5.0)	1 (2.8)	5 (4.3)
	Somewhat disagree	13 (16.7)	5 (21.7)	1 (6.7)	10 (17.2)	9 (15.5)	19 (16.4)	5 (13.9)	19 (16.4)
	Somewhat agree	35 (44.9)	15 (65.2)	4 (26.7)	28 (48.3)	26 (44.8)	54 (46.6)	17 (47.2)	54 (46.6)
	Agree	25 (32.1)	2 (8.7)	10 (66.7)	19 (32.8)	18 (31.0)	37 (31.9)	12 (33.3)	37 (31.9)
	Strongly agree	1 (1.3)	0	0	0	1 (1.7)	1 (0.9)	1 (2.8)	1 (0.9)
	**Total**	**78 (100)**	**23 (100)**	**15 (100)**	**58 (100)**	**58 (100)**	**1**1**6** (**1**0**0**	**36 (100)**	**116 (100)**
***If MBC was automated and available electronically with easy to interpret results, I would be more likely to use MBC.***	Strongly disagree	0	0	0	0	0	0	0	0
	Disagree	0	0	0	0	0	0	0	0
	Somewhat disagree	4 (5.1)	0	0	1 (1.7)	3 (5.2)	3 (3.8)	1 (2.8)	4 (3.4)
	Somewhat agree	9 (11.5)	3 (13.0)	1 (6.7)	5 (8.6)	8 (13.8)	6 (7.5)	7 (19.4)	13 (11.2)
	Agree	39 (50.0)	11 (47.8)	9 (60.0)	32 (55.2)	27 (46.6)	40 (50.0)	19 (52.8)	59 (50.9)
	Strongly agree	26 (33.3)	9 (39.1)	5 (33.3)	20 (34.5)	20 (34.5)	31 (38.8)	9 (25.0)	40 (34.5)
	**Total**	**78 (100)**	**23 (100)**	**15 (100)**	**58 (100)**	**58 (100)**	**80 (100)**	**36 (100)**	**116 (100)**
***I don’t know how to fit MBC into my current workflow because I am too busy.***	Strongly disagree	2 (2.6)	1 (4.3)	1 (6.7)	2 (3.4)	2 (3.4)	4 (5.0)	0	4 (3.4)
	Disagree	11 (11.4)	5 (21.7)	2 (13.3)	9 (15.5)	9 (15.5)	9 (11.3)	9 (25.0)	18 (15.5)
	Somewhat disagree	18 (23.1)	2 (8.7)	3 (20.0)	7 (12.1)	16 (27.6)	18 (22.5)	5 (13.9)	23 (19.8)
	Somewhat agree	23 (29.5)	9 (39.1)	4 (26.7)	21 (36.2)	15 (25.9)	25 (31.3)	11 (30.6)	36 (31.0)
	Agree	14 (17.9)	4 (17.4)	5 (33.3)	13 (22.4)	10 (17.2)	17 (21.3)	6 (16.7)	23 (19.8)
	Strongly agree	10 (12.8)	2 (8.7)	0	6 (10.3)	6 (10.3)	7 (8.8)	5 (13.9)	12 (10.3)
	**Total**	**78 (100)**	**23 (100)**	**15 (100)**	**58 (100)**	**58 (100)**	**80 (100)**	**36 (100)**	**116 (100)**

Regarding perceived feasibility of MBC, most surveyed clinicians agreed that they would use MBC if provided with proper training and resources, that they would use MBC to evaluate their own practice, and that they would be more likely to use MBC if it was automated or delivered via an electronic platform. The belief that electronic delivery would promote feasibility was also reflected in the qualitative interviews. A clinician from Fengxian center stated:

*“...it’s like more electronic and more easy, convenient. Better to have records saved, that can be accessed at any time, which may be more convenient to both doctors and patients. Also the records need to be improved, better to have some curve figure to show the results to make it more straightforward.” (FX07)*

Clinicians noted that using paper measures with standard MBC might be burdensome but that electronic delivery might help to simplify and streamline the process:

*“I don't necessarily have time to do the measures. If I have to do a self-rating scale, it is not convenient as well. Because I need to give the measures in paper to the patient and he might have to record the result in the computer at the end, and the workload would be a little bit heavy” (HK09).*

Perspectives among surveyed clinicians regarding the perceived willingness of their patients to complete measures were varied, as described in Table 4. Patient willingness and capacity emerged from the clinician interviews as a potential barrier to MBC implementation. As stated by clinicians from Fengxian and Hongkou:

*“...the biggest challenge is that patients may not be willing to collaborate, not willing to do it” (FX05).*

*“I think that patients should be able to accept it first. For example, it would be easily accepted in the inpatient ward. However in the outpatient clinics, there are many patients, they will not have enough patience for everyone doing the MBC” (HK08)*

Additional potential barriers were also raised:

*“Literacy level, age, and other factors need to be suitable for completing the measures” (FX01)*

Clinicians also expressed concern that depression symptoms, particularly among patients with severe depression, would affect their ability to complete the measures. They stated that measures must be simple, relatively brief and easy to understand.

*“Then there are some patients who may be resistant to the measure because of their illness. Since the patient is depressed, he is not interested in anything, he is not willing to touch anything, so that maybe creates a little problem when using the measures”. (HK07)*

Regarding whether MBC would be easy to integrate into their current workflow, a majority agree, as shown in Table 4, however disagreement is somewhat higher among SMHC clinicians. In qualitative interviews, workload and workflow emerged as potential barriers to MBC implementation. Clinicians from all MHCs identified time and number of patients as a major feasibility barrier:

*“...if [we] compare the MBC with our usual outpatient clinic, the clinician may not have enough time to meet with all the patients if they all used MBC, since we have roughly 40 to 50 patients in one single morning. MBC requires evaluating the patient and discussing with the patient his condition change, which is time consuming.” (HK02)*

Three people mentioned concern with patients having to pay out of pocket for measures, such as this interviewee from SMHC:

*“At present, we mainly use some measures that needed to be charged. During the follow-up appointment, most of the patients will consider the measures useless. “I don’t have to spend this money, I am already recovered” (SH01).*

Several strategies were recommended that might mitigate feasibility barriers, including those related to workflow and time. Some clinicians suggested allocating a designated staff person, such as a nurse or receptionist, to assist patients with measures as they perceived the demand would be too onerous for psychiatrists. They also suggested that a team-based approach that would facilitate MBC implementation should be mandated by management. Booking appointments specifically dedicated to MBC was suggested as a means of improving feasibility. These mechanisms to support MBC delivery are reflected in the following quote:

*“One [thing that is needed] is policy support. Personnel needs to be arranged. When patients come here, before seeing patients, someone needs to take them to do the measures, right? Communicate with doctors. Or the nurses or social workers can see the patients first, and send the completed measures to doctors. That’s good, I think. It needs a specific person to get it done, before the medical appointment.” (HK04)*

Separate rooms for patients to complete the measures were also recommended. A clinician from Hongkou stated:

*“…It is better to have a private, independent and quiet environment, so that the clinician can have a more detailed communication with the patient. This enables patients to make an objective assessment without reservation,” (HK03)*

Another clinician from SMHC reflected that this would be easier to implement in major urban hospital settings:

*“We can do this here, but I don't know if we can still do it elsewhere, because there is no psychological measurement room or assessors in the minor cities” (SH05)*

### Acceptability and perceived barriers and facilitators to use of enhanced MBC (eMBC)

Subsequent to asking about the use of standard MBC, we asked clinicians to rate their level of agreement about the acceptability of using eMBC based on a brief vignette describing the use of eMBC in a clinical setting. Responses are described in Table 5:

**Table 5:** Acceptability of use of eMBC

Survey Question	Response	Location			Age		Gender		
		SMHC: n (%)	HKMC: n (%)	FXMC: n (%)	18–39 years	40–59 years	F	M	Total: n (%)
**A mobile phone app, as described above, will make it easier to use MBC.**	Strongly disagree	0	0	0	0	0	0	0	0
	Disagree	0	1 (4.3)	0	0	1 (1.7)	1 (1.3)	0	1 (0.9)
	Somewhat disagree	2 (2.6)	2 (8.7)	0	2 (3.4)	2 (3.4)	2 (2.5)	2 (5.6)	4 (3.4)
	Somewhat agree	14 (17.9)	2 (8.7)	3 (20.0)	11 (19.0)	8 (13.8)	12 (15.0)	7 ()19.4)	19 (16.4)
	Agree	46 (59.0	15 (65.2)	7 (46.7)	32 (55.2)	36 (62.1)	46 (57.5)	22 (61.1)	68 (58.6)
	Strongly agree	16 (20.5)	3 (13.)	5 (33.3)	13 (22.4)	11 (19.0)	19 (23.8)	5 (13.9)	24 (20.7)
	**Total**	**78 (100)**	**23 (100)**	**15 (100)**	**58 (100)**	**58 (100)**	**80 (100)**	**36 (100)**	**116 (100)**
**I am willing to use a mobile phone app with my patients for MBC.**	Strongly disagree	1 (1.3)	0	0	0	1 (1.7)	0	1 (2.8)	1 (0.9)
	Disagree	1 (1.3)	0	0	1 (1.7)	0	0	1 (2.8)	1 (0.9)
	Somewhat disagree	7 (9.0)	2 (8.7)	0	3 (5.2)	6 (10.3)	6 (7.5)	3 (8.3)	9 (7.8)
	Somewhat agree	15 (19.2)	5 (21.7)	3 (20.0)	11 (19.0)	12 (20.7)	16 (20.0)	7 (19.4)	23 (19.8)
	Agree	41 (52.6)	10 (43.5)	10 (66.7)	34 (58.6)	27 (46.6)	41 (51.2)	20 (55.6)	61 (52.6)
	Strongly agree	13 (16.7)	5 (21.7)	2 (13.3)	9 (15.5)	11 (19.0)	16 (20.0	4 (11.1)	20 (17.2)
	**Total**	**78 (100)**	**23 (100)**	**15 (100)**	**58 (100)**	**57 (98.3)**	**79 (98.0)**	**36 (100)**	**116 (100)**
**My patients will find it easy to use a mobile phone app, as described above.**	Strongly disagree	0	0	0	0	0	0	0	0
	Disagree	0	2 (8.7)	2 (13.3)	3 (5.2)	1 (1.7)	3 (3.8)	1 (2.8)	4 (3.4)
	Somewhat disagree	9 (11.5)	4 (17.4)	0	4 (6.9)	9 (15.5)	10 (12.5)	3 (8.3)	13 (11.2)
	Somewhat agree	24 (30.8)	10 (43.5)	5 (33.3)	22 (37.9)	17 (29.3)	26 (32.5)	13 (36.1)	39 (33.6)
	Agree	38 (48.7)	7 (30.4)	8 (53.3)	27 (46.6)	26 (44.8)	38 (47.5)	15 (41.7)	53 (45.7)
	Strongly agree	7 (9.0)	0	0	2 (3.4)	5 (8.6)	3 (3.8)	4 (11.1)	7 (6.0)
	**Total**	**78 (100)**	**23 (100)**	**15 (100)**	**58 (100)**	**58 (100)**	**80 (100)**	**36 (100)**	**116 (100)**
**My patients would be willing to use a mobile phone app to track their outcomes.**	Strongly disagree	0	0	0	0	0	0	0	0
	Disagree	2 (2.6)	2 (8.7)	1 (6.7)	2 (3.4)	3 (5.2)	5 (6.3)	0	5 (4.3)
	Somewhat disagree	7 (9.0)	4 (17.4)	1 (6.7)	8 (13.8)	4 (6.9)	11 (13.8)	1 (2.8)	12 (10.3)
	Somewhat agree	28 (35.9)	11 (47.8)	3 (20.0)	20 (34.5)	22 (37.9)	26 (32.5)	16 (44.4)	42 (36.2)
	Agree	33 (42.3)	6 (26.1)	10 (66.7)	26 (44.8)	23 (39.7)	34 (42.5)	15 (41.7)	49 (42.2)
	Strongly agree	8 (10.3)	0	0	2 (3.4)	6 (10.3)	4 (5.0)	4 (11.1)	8 (6.9)
	**Total**	**78 (100)**	**23 (100)**	**15 (100)**	**58 (100)**	**58 (100)**	**80 (100)**	**36 (100)**	**116 (100)**

Qualitative interview results provided further information on clinicians’ views of the potential positive and negative effects that eMBC could have on patient care.

Many clinicians stated that they believe eMBC would be more comprehensive, efficient and convenient than current practice. eMBC could improve treatment plans for patients and improve symptom tracking. A clinician from Hongkou stated:

*“In this way, it’ll save doctors some time. Doctors can have deeper communication with patients on some highlights of their issues. Based on the assessments results, we may skip some unimportant things, and avoid some ineffective consultation.” (HK01)*

Clinicians also acknowledged that eMBC would help to avoid some of the challenges identified in relation to standard MBC. A clinician from Fengxian stated:

*“It’s also mobile and portable, and hence the tool itself is very convenient to patients. Patients may complete when they have time, and they do not have to do it during their wait for the appointments. Hence its usability and accessibility is very good”.* (SH09)

Additionally, clinicians believed that there are multiple ways in which eMBC could improve patients’ engagement with and adherence to treatment. Firstly, using the patient’s input as a valuable part of the treatment plan can strengthen the therapeutic alliance:

*“I think it’d be that patients’ feelings would be better respected in this way, so that there’d be a better relationship with patients and doctors. Their adherence to medications and treatments may be improved*” (FX09)

With improved patient-clinician understanding, the clinician can offer a more holistic and individualized perspective on the patient’s experiences and priorities regarding treatment, as stated by clinician from Hongkou and SMHCs:

*“I think it’s more targeted. And it’ll be easier for us to understand patients, such as what treatments they prefer, or which issues they are more concerned with and want to solve first. When we make decisions, we’ll pay more attention to these things”* (HK05).

*“There is definitely benefits. ...Lot of studies had confirmed that the treatment of depression will be better if the doctor's treatment plan is in line with the patient's own wishes”.* (SH 03)

Clinicians also noted that a patient’s enhanced understanding of their condition could also provide positive effects including stigma reduction:

*“In the process, there is not only an assessment, but also health education and the understanding of disease knowledge, which can help patients to eliminate fear and shame of depression. Then they can find out from the assessment which aspects have a more positive change, thus accumulating confidence in the treatment” (SH10)*

Though clinicians believed eMBC offers many avenues to improve patient care, some also spoke to the importance of contextualizing eMBC results with other forms of clinical data and processes:

*“The measurement in MBC should be comprehensive, including the psychiatric exams, i.e. doctor’s assessment, feedback from families, etc. All these together are called measurement-based. If it’s only based on self-report, I don’t think it’s measurement-based. It’s just one-sided. Hence, measurement-based care is good, but if you only use this one tool, there must be bias”. (SH08)*

Additionally, they noted that eMBC should not be viewed as a replacement for usual clinical contact:

*“Of course, no assessments/measures can replace the actual communication between doctors and patients, including collecting information such as their body language, tone, or even their smell, etc., which cannot be assessed by the paper or mobile measures/scales. Hence, (the measures/scales) as a supplement” (SH09)*

Although clinicians showed a high level of agreement about their willingness to use eMBC and their perception that it would make MBC easier to use, they also identified several potential barriers. A clinician from Fengxian questioned how the formality of a typical consultation would be maintained as clinicians and patients communicate within an app:

*“However in fact, we still hope to provide formal diagnosis, formal treatments and formal suggestions in the medical authorities, which have better qualifications for medical practice, instead of providing some suggestions in the chats. The patients may consider those chats as doctors’ instructions, which may be not formal*.” *(FX09)*

Some saw enhancing the role of patients in treatment decision-making as a threat to the status quo where the clinician’s knowledge is unquestioned:

*“What he considers the treatments may sometimes contradict to our medical knowledge. He will apply those treatment strategies, and thinks that this is what he needs most. In fact, this is not the best strategy. This is a challenge”. (HK09)*

*“I don’t like it. I feel that if you let patients to use the mobile phone by themselves, then their trust for doctors will go down. They’ll feel you are not useful, and they are completing all things by themselves”. (HK06)*

Clinicians also questioned patients’ capacity to complete self-report measures as a consequence of depressive symptoms, including low motivation, functional ability and cognitive impairment. One Hongkou clinician stated:

*“There is no big challenge for clinicians, I can use cell phone apps, and be able to see the patients’ scores. I don't think there is any big challenge for me. But for the patient, the challenge is that once his condition worsens and there is severe depression, it may not be possible to complete the eMBC”. (HK02)*

Others questioned the benefits of enhancing patient knowledge about depression and treatment options:

*“For example, some anti-depressants may have some side effects. If you tell them about these side effects, patients may have some concerns. Another thing is that if patients know too much about their medical conditions, it will affect their moods?” (FX06)*

A clinician for SMHC states: “I mean, sometimes it may not be good for them to know too much”. (SH06).

In interviews, clinicians also raised several concerns about the ability of patients to accurately complete measures delivered via an app. This included the possibility that a patients’ family might complete the measures on their behalf, meaning that the results might not accurately reflect the patient’s condition.

*“They may randomly choose an answer, which is meaningless. That’s why self-report should be used together with administered report. Self-report is not always totally reliable, and it can just be used as a reference. At the beginning, they may take it serious, and do it carefully, but after a while, they become unwilling to do it”. (SH08)*

Similarly to standard MBC, clinicians also raised concerns about workload. They worried that eMBC would require more explanation and would generate more questions from patients that would infringe on clinicians’ time:

*“I think it’s time consuming to let patients scan the QR code, and the process… to download this thing using the QR code to explain it.. I mean, the work of explaining it to clinical patients, and to promote/implement it, would be a challenge”. (SH02)*

Some also questioned whether the nature of eMBC and the use of mobile apps would impinge on their personal time, requiring them to engage with patients outside of regular clinic hours:

*“... if it’s like Wechat and when the patients add you on it, they might occupy your personal time (for consultation). I think the word “occupy” in this situation is appropriate.” (FX03)*

Literacy, including digital and health literacy, was identified as a potential barrier, especially for seniors, people with lower levels of formal education, people with disabilities or people living in rural or remote areas:

*“Some seniors may not know how to use a mobile phone, and another [challenge] is that if their literacy level is not high enough, they may not understand it, or may not understand some questions, right?” (FX02)*

One interviewee also spoke to concerns about physician capacity to utilize apps, although there was no variation in responses about eMBC acceptability by age group among surveyed clinicians:

*“For some older clinicians, the challenges might be such as the application of the mobile phone is not familiar which makes them unable to use it” (HK09)*

Clinicians also believed that privacy and security considerations might deter patients from using eMBC:

*“I think the issue for this kind of tool is data confidentiality. For patients’ privacy, they may have concerns about who has the access to data and who will know their information, what this information will be used for”. (SH09)*

In addition to reporting the perceived positive and negative considerations for implementing eMBC, several clinicians also spoke to their perceptions of system-level facilitators for eMBC, including the need for marketing and promotion of the intervention:

*“There must be publicity for the two treatments since not everyone can accept these care right away. The treatments are very good but not every good thing can be accepted by everyone. Therefore, publicity is important so as to let the patients and the clinicians better understand the treatment methods. What do MBC/eMBC mean to the treatment? What outcomes will they bring to the clinical practice? What are the difference between the two? The clinician and the patient need to accept MBC and eMBC from the bottom of their heart so that it is easier to implement and market in the clinical practice”. (HK08)*

Provision of training for both patients and clinicians was seen as essential, as described by a clinician from Hongkou, who stated:

*“For initial visits, we can arrange specific staff to provide health education (for clinicians) including training on measures. Professional assistance is needed to do it”* and *“I think for the first time using the measures, there should be some health education to make sure patients can understand the terms correctly”. (HK06)*

### Mixed methods results: patients

We surveyed and conducted focus groups with patients to assess and understand their current use of Internet and mobile technology, their use of the Internet for health information and support, and the acceptability of standard and eMBC. *N* = 301 patients responded to an online survey (SMHC *n* = 151, HKMC *n* = 88, FXMC *n* = 62). We conducted six focus groups and stratified by age (18–44 years and 45 years and over) at FXMC and HKMC. Two focus groups of mixed ages were held at SMHC, with *n* = 4 and *n* = 2 participants in each. At FXMC, *n* = 3 people participated in the younger group and n = 2 people in the older group, while at HKMC there were n = 4 in the older and n = 4 in the younger group.

### Patient demographics

Demographic information for patient survey respondents is displayed in Table 6:

**Table 6:** Patient demographics

Survey Question	Response	SMHC: n (%)	HKMC: n (%)	FXMC: n (%)	Total: n (%)
**Age**	18–29	99 (65.6)	2 (2.3)	8 (11.4)	109 (35.3)
	30–39	30 (19.9)	8 (9.1)	13 (18.6)	51 (16.5)
	40–49	10 (6.6)	12 (13.6)	11 (15.7)	33 (10.7)
	50–59	6 (4.0)	16 (18.2)	21 (30)	43 (13.9)
	60–69	5 (3.3)	40 (45.5)	15 (21.4)	60 (19.4)
	70+	1 (0.7)	10 (11.4)	2 (2.9)	13 (4.2)
	**Total**	**151 (100)**	**88 (100)**	**70 (100)**	**309 (100)**
**Gender**	Female	109 (72.2)	64 (72.7)	45 (64.3)	218 (70.6)
	Male	40 (26.5)	23 (26.1)	25 (35.7)	88 (28.5)
	Prefer not to answer	2 (1.3)	1 (1.1)	0 (0.0)	3 (1.0)
	**Total**	**151 (100)**	**88 (100)**	**70 (100)**	**309 (100)**
**Are you currently a resident of Shanghai**	Yes	98 (64.9)	86 (97.7)	59 (84.3)	243 (78.6)
	No	53 (35.1)	2 (2.3)	11 (15.7)	66 (21.4)
	**Total**	**151 (100)**	**88 (100)**	**70 (100)**	**309 (100)**
**Please select the type of area you live in**	Urban area	110 (72.8)	82 (93.2)	6 (8.6)	198 (64.1)
	Suburban area	39 (25.8)	5 (5.7)	51 (72.9)	95 (30.7)
	Rural area	2 (1.3)	1 (1.1)	13 (18.6)	16 (5.2)
	**Total**	**151 (100)**	**88 (100)**	**70 (100)**	**309 (100)**
**Please select the highest level of education you have obtained**	Primary school	0	1 (1.1)	5 (7.1)	6 (1.9)
	Middle school	8 (5.3)	27 (30.7)	20 (28.6)	55 (17.8)
	High school	25 (16.6)	35 (39.8)	26 (37.1)	86 (27.8)
	Undergraduate degree	78 (51.7)	14 (15.9)	12 (17.1)	104 (33.7)
	Vocational school	13 (8.6)	2 (2.3)	5 (7.1)	20 (6.5)
	Post-graduate or professional degree	24 (15.9)	4 (4.5)	0 (0.0)	28 (9.1)
		3 (2.0)	5 (5.7)	2 (2.9)	10 (3.2)
	**Total**	**151 (100)**	**88 (100)**	**70 (100)**	**309 (100)**
**Please select your current employment status**	Employed full-time	59 (39.1)	22 (25.0)	22 (31.4)	103 (33.3)
	Employed part-time	3 (2.0)	0	2 (2.9)	5 (1.6)
	Self employed	9 (6.0)	3 (3.4)	6 (8.6)	18 (5.8)
	At-home parent	4 (2.6)	0	1 (1.4)	5 (1.6)
	Not employed	14 (9.3)	2 (2.3)	6 (8.6)	22 (7.1)
	Retired	9 (6.0)	60 (68.2)	26 (37.1)	95 (30.7)
	Student	49 (32.5)	1 (1.1)	5 (7.1)	55 (17.8)
	Other (fill in)	4 (2.6)	0	2 (2.9)	6 (1.9)
	**Total**	**151 (100)**	**88 (100)**	**70 (100)**	**309 (100)**

There is variation in the age of patient respondents between SMHC, where a majority (65.6%) are under 30 years old, and at the other two health centers where patients respondents are much older. The predominance of younger participants from SMHC may be due to the nature of the hospital, as it is one of the top tier psychiatric hospitals in China and attracts patients from across the country, many of whom are young. Younger patients may also be more willing to complete an online survey. At HKMC, 63.7% of respondents are between 50 and 69 years, and at FXMC 50% are between 50 and 69 years. The majority of respondents (72.4%) across all health centers are female, which is consistent with patient demographics at all centers. Though a majority (78.4%) are Shanghai residents, 21.6% indicate that they are not, and have likely traveled to Shanghai from elsewhere for mental health services. A majority of patients are educated at the high school (27.2%) or undergraduate (34.6) level, though in Hongkou and Fengxian 30.7 and 27.4% respectively report high school as their highest level of educational attainment. The majority of patient respondents are currently employed full-time (33.6%), retired (30.6%) or students (18.3%), with the highest number (68.2%) of retirees in Hongkou district and the highest number of students (32.5%) among SMHC respondents.

### Patient use of internet and Mobile technology

We asked patients to respond to survey questions about their current use of the Internet and mobile technology, as displayed in Table 7:

**Table 7:** Patient use of Internet and Mobile Technology

Survey	Response	Location			Age		Gender			Total: n (%)
		SMHC: n (%)	HKMC: n (%)	FXMC: n (%)	18–39 years	40+ years	F	M	Unknown	
**How frequently do you use the Internet?**	Many times a day	137 (90.7)	68 (77.3)	49 (70.0)	146 (91.3)	108 (72.5)	182 (83.5)	70 (79.5)	2 (66.7)	254 (82.2)
	Once a day	8 (5.3)	7 (8.0)	5 (7.1)	5 (3.1)	15 (10.1)	11 (5.0)	9 (10.2)	0 (0.0)	20 (6.5)
	Once every few days	1 (0.7)	4 (4.5)	1 (1.4)	2 (1.3)	4 (2.7)	2 (0.9)	4 (4.5)	0 (0.0)	6 (1.9)
	Once a week	3 (2.0)	0	1 (1.4)	2 (1.3)	2 (1.3)	2 (0.9)	2 (2.3)	0 (0.0)	4 (1.3)
	A few times a month	1 (0.7)	2 (2.3)	1 (1.4)	2 (1.3)	2 (1.3)	3 (1.4)	0 (0.0)	1 (33.3)	4 (1.3)
	Rarely or not at all	1 (0.7)	7 (8.0)	13 (18.6)	3 (1.9)	18 (12.1)	18 (8.3)	3 (3.4)	0 (0.0)	21 (6.8)
	**Total**	**151 (100)**	88 (100)	**70 (100)**	**160 (100)**	**149 (100)**	**218 (100)**	**88 (100)**	**3 (100)**	**309 (100)**
**Which statement most accurately describes your access to a Smartphone?**	I own my own Smartphone	149 (98.7)	81 (92.0)	62 (88.6)	158 (98.8)	134 (89.9)	204 (93.6)	85 (96.6)	3 (100)	292 (94.5)
	I share a Smartphone with family members or friends	1 (0.7)	2 (2.3)	1 (1.4)	1 (0.6)	3 (2.0)	2 (0.9)	2 (2.3)	0 (0.0)	4 (1.3)
	I do not own or have access to a Smartphone	1 (0.7)	2 (2.3)	6 (8.6)	1 (0.6)	8 (5.4)	8 (3.7)	1 (1.1)	0 (0.0)	9 (2.9)
	Other	0	3 (3.4)	1 (1.4)	0 (0.0)	4 (2.7)	4 (1.8)	0 (0.0)	0 (0.0)	4 (1.3)
	**Total**	**151 (100)**	**88 (100)**	**70 (100)**	**160 (100)**	**149 (100)**	**218 (100)**	**8 (100)**	**3 (100)**	**309 (100)**
**If you selected i or ii in the previous question, how long have you been using a Smartphone?**	Less than 1 year	3 (2.0)	4 (4.5)	5 (7.9)	5 (3.1)	7 (5.1)	6 (2.9)	6 (6.9)	0 (0.0)	12 (4.1)
	2–3 years	13 (8.6)	11 (12.5)	10 (15.9)	8 (5.0)	26 (19.0)	26 (12.6)	8 (9.2)	0 (0.0)	34 (11.5)
	3–5 years	20 (13.2)	29 (33.0)	14 (22.2)	22 (13.8)	41 (29.9)	43 (20.9)	20 (23.0)	0 (0.0)	63 (21.3)
	More than 5 years	107 (70.8)	38 (43.2)	32 (50.8)	118 (74.2)	59 (43.1)	124 (60.2)	50 (57.5)	3 (100)	177 (59.8)
	Missing	8 (5.3)	6 (6.8)	2 (3.2)	6 (3.8)	4 (2.9)	7 (3.4)	3 (3.4)	0 (0.0)	10 (3.4)
	**Total**	**151 (100)**	**88 (100)**	**63 (100)**	**159 (100)**	**137 (100)**	**206 (100)**	**87 (100)**	**3 (100)**	**296 (100)**
**How do you usually access the Internet?**	I only access the Internet on my Smartphone	31 (20.5)	39 (44.3)	29 (41.4)	26 (16.3)	73 (49.0)	70 (32.1)	29 (33.0)	0 (0.0)	99 (32.0)
	I use both my Smartphone and a computer to access the Internet	117 (77.5)	34 (38.6)	26 (37.1)	133 (83.1)	44 (29.5)	122 (56.0)	52 (59.1)	0 (0.0)	177 (57.3)
	I only access the Internet on a computer	2 (1.3)	2 (2.3)	0 (0.0)	0 (0.0)	4 (2.7)	3 (1.4)	1 (1.1)	0 (0.0)	4 (1.3)
	I rarely access the Internet	1 (0.7)	11 (12.5)	3 (4.3)	1 (0.6)	14 (9.4)	12 (5.5)	3 (3.4)	0 (0.0)	15 (4.9)
	I never access the Internet	0	2 (2.3)	12 (17.1)	0 (0.0)	14 (9.4)	11 (5.0)	3 (3.4)	0 (0.0)	14 (4.5)
	**Total**	**151 (100)**	**88 (100)**	**70 (100)**	**160 (100)**	**149 (100)**	**218 (100)**	**88 (100)**	**3 (100)**	**309 (100)**
**How familiar are you with using mobile apps on your Smartphone?**	Very familiar	110 (72.8)	29 (33.0)	22 (31.4)	124 (77.5)	37 (24.8)	111 (50.9)	49 (55.7)	1 (33.3)	161 (52.1)
	Somewhat familiar	39 (25.8)	49 (55.7)	34 (48.6)	35 (21.9)	87 (58.4)	89 (40.8)	32 (36.4)	1 (33.3)	122 (39.5)
	Not at all familiar	1 (0.7)	6 (6.8)	9 (12.9)	0 (0.0)	16 (10.7)	11 (5.0)	5 (5.7)	0 (0.0)	16 (5.2)
	Not applicable	1 (0.7)	4 (4.5)	5 (7.1)	1 (0.6)	9 (6.0)	7 (3.2)	2 (2.3)	1 (33.3)	10 (3.2)
	**Total**	**151 (100)**	**88 (100)**	**70 (100)**	**160 (100)**	**149 (100)**	**218 (100)**	**88 (100)**	**3 (100)**	**309 (100)**
**How familiar are you with the WeChat app?**	Very familiar	125 (82.8)	45 (51.1)	34 (48.6)	131 (81.9)	73 (49.0)	142 (65.1)	61 (69.3)	1 (33.3)	204 (66.0)
	Somewhat familiar	24 (15.9)	36 (40.9)	27 (38.6)	27 (16.9)	60 (40.3)	63 (28.9)	23 (26.1)	1 (33.3)	87 (28.2)
	Not at all familiar	0	5 (5.7)	3 (4.3)	0 (0.0)	8 (5.4)	6 (2.8)	2 (2.3)	0 (0.0)	8 (2.6)
	Not applicable	2 (1.3)	2 (2.3)	6 (8.6)	2 (1.3)	8 (5.4)	7 (3.2)	2 (2.3)	1 (33.3)	10 (3.2)
	T**otal**	**151 (100)**	**88 (100)**	**70 (100**)	**160 (100)**	**149 (100)**	**218 (100)**	**88 (100)**	**3 (100)**	**309 (100)**
**If you selected i or ii for question 2.6, how often do you use WeChat?**	Many times a day	140 (92.7)	68 (77.3)	50 (71.4)	150 (94.9)	108 (81.2)	185 (90.2)	71 (84.5)	2 (100)	258 (88.7)
	Once a day	4 (2.6)	8 (9.1)	7 (10)	3 (1.9)	16 (12.0)	11 (5.4)	8 (9.5)	0 (0.0)	19 (6.5)
	Once every few days	2 (1.3)	2 (2.3)	3 (4.3)	4 (2.5)	3 (2.3)	4 (2.0)	3 (3.6)	0 (0.0)	7 (2.4)
	Once a week	1 (0.7)	0	0 (0.0)	0 (0.0)	1 (0.8)	1 (0.5)	0 (0.0)	0 (0.0)	1 (0.3)
	A few times a month	1 (0.7)	1 (1.1)	0 (0.0)	1 (0.6)	1 (0.8)	1 (0.5)	1 (1.2)	0 (0.0)	2 (0.7)
	Rarely or not at all	1 (0.7)	2 (2.3)	1 (1.4)	0 (0.0)	4 (3.0)	3 (1.5)	1 (1.2)	0 (0.0)	4 (1.4)
	Missing									
	Total	151 (100)	88 (100)	61 (100)	158 (100)	133 (100)	205 (100)	84 (100)	2 (100)	291 (100)

A majority (82.1%) of patient respondents across all MHCs indicate that they use a smartphone many times a day, however it is notable that among respondents from FXMC 21.0% indicated that they use one rarely or not at all. 94.4% of patient respondents own a smartphone, however 9.7% of FXMC respondents indicated that they do not own or have access to one. Of patient respondents who own a smartphone, the majority have been using one for more than a year, as show in Table 7. Among patient respondents from SMHC, 70.8% have been using a smartphone for more than 5 years. Regarding frequency of Internet access, across all centers 31.6% of patients respondents use the Internet only on their smartphone, 57.8% use both a smartphone and computer to access the Internet. Among HKMC respondents, 12.5% indicate that they rarely use the Internet, and at FXMC 17.7% indicate that they never use the Internet.

Across all centers, patient familiarity with using mobile apps is high. While fewer than 1% of SMHC respondents indicated that they are not at all familiar or selected not applicable, 11.3% of HKMC and 19.4% of FXMC respondents selected not at all or not applicable. Familiarity was somewhat lower among patients over 40 years, with 58.4% indicating they are somewhat familiar with using mobile apps, while 77.5% of patients under 40 indicated they are very familiar. A majority of patients across all MHCs are familiar with WeChat, though there is some variation in age, with 81.9% of patients under 40 years indicating they are very familiar compared with 49.0% of those over 40. Among WeChat users, 84.1% of patients across all health centers use the app many times per day.

### Patient use of the internet for health information and support

We asked patients to indicate the degree to which they use the Internet for health information and support, as described in Table 8:

**Table 8:** Patient use of Internet for health information and support

Survey	Response	Location			Age		Gender			Total: n (%)
		SMHC: n (%)	HKMC: n (%)	FXMC: n (%)	18–39 years	40+ years	F	M	Unknown	
**I access information about general health issues on the Internet**	Often	64 (42.4)	33 (37.5)	26 (37.1)	70 (43.8)	53 (35.6)	83 (38.1)	39 (44.3)	1 (33.3)	123 (39.8)
	Sometimes	80 (53.0)	45 (51.1)	22 (31.4)	81 (50.6)	66 (44.3)	107 (49.1)	38 (43.2)	2 (66.7)	147 (47.6)
	Never	7 (4.6)	10 (11.4)	22 (31.4)	9 (5.6)	30 (20.1)	28 (12.8)	11 (12.5)	0 (0.0)	39 (12.6)
	**Total**	**151 (100)**	**88 (100)**	**70 (100)**	**160 (100)**	**149 (100)**	**218 (100)**	**88 (100)**	**3 (100)**	**309 (100)**
**I access information about mental health issues on the Internet**	Often	47 (31.1)	30 (34.1)	22 (31.4)	54 (33.8)	45 (30.2)	68 (31.2)	30 (34.1)	1 (33.3)	99 (32.0)
	Sometimes	92 (60.9)	45 (51.1)	27 (38.6)	92 (57.5)	72 (48.3)	117 (53.7)	45 (51.1)	2 (66.7)	164 (53.1)
	Never	12 (7.9)	13 (14.8)	21 (30)	14 (8.8)	32 (21.5)	33 (15.1)	13 (14.8)	0 (0.0)	46 (14.9)
	**Total**	**151 (100)**	**88 (100)**	**70 (100)**	**160 (100)**	**149 (100)**	**218 (100)**	**88 (100)**	**3 (100)**	**309 (100)**
**If you selected i or ii to questions 3.1 and/ or 3.2,what types of Internet sources have you used to access health information (please select all that apply).**	Official websites	56 (38.4)	34 (42.5)	17 (33.3)	57 (37.7)	50 (40.7)	73 (37.2)	32 (41.0)	2 (66.7)	107 (38.6)
	Online discussion forums	45 (30.8)	14 (17.5)	8 (15.7)	48 (31.2)	19 (15.4)	46 (23.5)	20 (25.6)	1 (33.3)	67 (24.2)
	Online or mobile apps	63 (43.2)	24 (30.0)	25 (49.0)	73 (47.4)	39 (31.7)	72 (36.7)	38 (48.7)	2 (66.7)	112 (40.4)
	Social media	67 (35.9)	41 (51.3)	23 (45.1)	72 (46.8)	59 (48.0)	93 (47.4)	38 (48.7)	0 (0.0)	131 (47.3)
	Other (fill in)	11 (7.5)	8 (10.0)	4 (7.8)	11 (7.1)	12 (9.8)	19 (9.7)	3 (3.8)	1 (33.3)	23 (8.3)
	Missing	2 (1.4)	0 (0.0)	0 (0.0)	2 (1.3)	0 (0.0)	2 (1.0)	0 (0.0)	0 (0.0)	2 (0.7)
	**Total**	**146 (100)**	**80 (100)**	**51 (100)**	**154 (100)**	**123 (100)**	**196 (100)**	**78 (100)**	**3 (100)**	**277 (100)**
**If you selected i or ii to questions 3.1 and/ or 3.2,what types of information do you generally search for? (Choose all that apply)**	Information about medications	58 (39.8)	42 (52.5)	18 (35.3)	62 (40.2)	56 (45.5)	85 (43.4)	30 (38.5)	3 (100)	118 (52.3)
	Information on how to cope with or manage symptoms	82 (56.2)	36 (45.0)	15 (29.4)	84 (54.5)	49 (39.8)	94 (48.0)	36 (46.2)	3 (100)	133 (48.0)
	General information about health and/ or mental health problems	119 (81.5)	45 (56.3)	34 (66.7)	121 (78.6)	77 (62.6)	141 (71.9)	54 (69.2)	3 (100)	198 (71.5)
	Information about general health and/ or mental health services	62 (42.5)	36 (45.0)	19 (37.3)	63 (40.3)	54 (43.9)	88 (44.9)	27 (34.6)	2 (66.7)	117 (42.2)
	Other (fill)	4 (2.7)	7 (8.8)	3 (5.9)	6 (39.0)	8 (6.5)	12 (6.1)	2 (2.6)	0 (0.0)	14 (5.1)
	**Total**	**146 (100)**	**80 (100)**	**51 (100)**	**154 (100)**	**123 (100)**	**196 (100)**	**78 (100)**	**3 (100)**	**277 (100)**
**I feel confident in my ability to use the Internet to find health and mental health information**	Strongly Disagree	1 (0.7)	1 (1.1)	0 (0.0)	1 (0.6)	1 (0.7)	2 (0.9)	0 (0.0)	0 (0.0)	2 (0.6)
	Disagree	8 (5.3)	4 (4.5)	6 (8.6)	6 (3.8)	12 (8.1)	10 (4.6)	8 (9.1)	0 (0.0)	18 (5.8)
	Undecided	56 (37.1)	20 (22.7)	31 (44.3)	63 (39.4)	44 (29.5)	74 (33.9)	32 (36.4)	1 (33.3)	107 (34.6)
	Agree	77 (51.0)	61 (69.3)	30 (42.9)	79 (49.4)	89 (59.7)	122 (56.0)	44 (50.0)	2 (66.7)	168 (54.4)
	Strongly Agree	9 (6.0)	2 (2.3)	3 (4.3)	11 (6.9)	3 (2.0)	10 (4.6)	4 (4.5)	0 (0.0)	14 (4.5)
	**Total**	**151 (100)**	**88 (100)**	**70 (100)**	**160 (100)**	**149 (100)**	**218 (100)**	**88 (100)**	**3 (100)**	**309 (100)**
**I feel confident in my ability to tell the difference between high quality and low quality sources of health information on the Internet**	Strongly Disagree	0 (0.0)	1 (1.1)	0 (0.0)	0 (0.0)	1 (0.7)	1 (0.5)	0 (0.0)	0 (0.0)	1 (0.3)
	Disagree	9 (6.0)	4 (4.5)	7 (10)	9 (5.6)	11 (7.4)	13 (6.0)	7 (8.0)	0 (0.0)	20 (6.5)
	Undecided	57 (37.7)	33 (37.5)	31 (44.3)	64 (40.0)	57 (38.3)	93 (42.7)	26 (29.6)	2 (66.7)	121 (39.2)
	Agree	74 (49.0)	48 (54.5)	31 (44.3)	76 (47.5)	77 (51.7)	103 (47.2)	49 (55.7)	1 (33.3)	153 (49.5)
	Strongly Agree	11 (7.3)	2 (2.3)	1 (1.4)	11 (6.9)	3 (2.0)	8 (3.7)	6 (6.8)	0 (0.0)	14 (4.5)
	**Total**	**151 (100)**	**88 (100)**	**70 (100)**	**160 (100)**	**149 (100)**	**218 (100)**	**88 (100)**	**3 (100)**	**309 (100)**
**I feel confident in my ability to use health information from the Internet to make health decisions**	Strongly Disagree	0 (0.0)	1 (1.1)	0 (0.0)	0 (0.0)	1 (0.7)	1 (0.5)	0 (0.0)	0 (0.0)	1 (0.3)
	Disagree	26 (17.2)	4 (4.5)	8 (11.4)	27 (16.9)	11 (7.4)	22 (10.1)	16 (18.2)	0 (0.0)	38 (12.3)
	Undecided	62 (41.1)	35 (39.8)	35 (50)	67 (41.9)	65 (43.6)	100 (45.9)	30 (34.1)	2 (66.7)	132 (42.7)
	Agree	58 (38.4)	47 (53.4)	26 (37.1)	61 (38.1)	70 (47.0)	91 (41.7)	39 (44.3)	1 (33.3)	131 (42.4)
	Strongly Agree	5 (3.3)	1 (1.1)	1 (1.4)	5 (3.1)	2 (1.3)	4 (1.8)	3 (3.4)	0 (0.0)	7 (2.3)
	**Total**	**151 (100)**	**88 (100)**	**70 (100)**	**160 (100)**	**149 (100)**	**218 (100)**	**88 (100)**	**3 (100)**	**309 (100)**
**I have previously used resources available on the Internet (e.g. web-based or mobile apps) to support me with managing health conditions**	Yes	58 (38.4)	38 (43.2)	29 (41.4)	61 (38.1)	64 (43.0)	96 (41.7)	34 (38.6)	0 (0.0)	125 (40.5)
	No	74 (49.0)	31 (35.2)	23 (32.9)	79 (49.4)	49 (32.9)	81 (39.4)	40 (45.5)	2 (66.7)	128 (41.4)
	Not sure	19 (12.6)	19 (21.6)	18 (25.7)	20 (12.5)	36 (24.2)	41 (18.8)	14 (15.9)	1 (33.3)	56 (18.1)
	**Total**	**151 (100)**	**88 (100)**	**70 (100)**	**160 (100)**	**149 (100)**	**218 (100)**	**88 (100)**	**3 (100)**	**309 (100)**

As shown in Table 8, a majority of patients use the Internet to access general health information often (30.9%) or sometimes (47.8%). 4.6% of SMHC respondents, 11.4% of HKMC and 32.3% of FXMC patients indicate that they never use the Internet for health information. A majority of surveyed patients use the Internet for information specific to mental health, however among FXMC patients 30.0% state that they never use it, compared with 7.9% at SMHC and 14.8% at HKMC. Of the respondents who do use the Internet to search for mental health information, a variety of sources are accessed with little variation across mental health centers, as shown in Table 8.

In patient focus groups, participants described their use of the Internet for mental health information. A participant from an older focus group stated:

*“When I was just diagnosed with depression… I did research about depression. I searched on* *Sohu.com* *, with information about the symptoms of depression, how bad it will be with no treatments, and why.” (HKMC Older)*

A younger participant described the benefits they received from accessing online information:

*When I first identified depression, I stayed into the patient group chat for quite a long time. Then I gradually followed some official accounts on Wechat. There were so many official accounts with inspiring articles which related to psychology. They benefit me very much, I got a lot of help from them. (SMHC Younger)*

We also asked patients to indicate their level of perceived self-efficacy regarding the use of Internet resources for health decision-making and management. Regarding their confidence in using the Internet to find general and mental health related information a majority (55.5%) agreed though 34.2% were undecided, with results consistent across MHCs. In terms of their confidence in their ability to discern between high and low quality health information online, results were similar to the previous question. When asked about their confidence in using information found online to make health decisions, 43.5% agreed, 42.2% were undecided. There was more variation across levels of disagreement between MHC’s in this category, with 17.2% of SMHC patients disagreeing, while 4.5% from HKMC and 8.1% from FXMC disagreed. Finally, we asked patients to indicate whether they had previously used Internet sources to help manage their mental health conditions. 40.9% said yes, 40.9% said no, and 18.3% were not sure, with responses consistent across MHCs.

### Patient acceptability of MBC and eMBC

Both surveyed patients and focus group participants were provided with brief vignettes describing the use of standard in-clinic MBC and eMBC and were asked to respond to questions related to acceptability of each approach. Survey results of patient acceptability of standard MBC are displayed in Table 9:

**Table 9:** Patient acceptability of standard, in-clinic MBC

Survey	Response	Location			Age		Gender			Total: n (%)
		SMHC: n (%)	HKMC: n (%)	FXMC: n (%)	18–39 years	40+ years	F	M	Unknown	
**I would be willing to spend 5 min completing a questionnaire as described in the scenario above before each appointment with my doctor**	Strongly disagree	0 (0.0)	0 (0.0)	0 (0.0)	0 (0.0)	0 (0.0)	0 (0.0)	0 (0.0)	0 (0.0)	0 (0.0)
	Disagree	2 (1.3)	3 (3.4)	1 (1.4)	1 (0.6)	5 (3.4)	4 (1.8)	2 (2.3)	0 (0.0)	6 (1.9)
	Somewhat disagree	5 (3.3)	14 (15.9)	6 (8.6)	8 (5.0)	17 (11.4)	19 (8.7)	5 (5.7)	1 (33.3)	25 (8.1)
	Somewhat agree	23 (15.2)	12 (13.6)	12 (17.1)	25 (15.6)	22 (14.8)	30 (13.8)	16 (18.2)	1 (33.3)	47 (15.2)
	Agree	98 (64.9)	56 (63.6)	48 (68.6)	101 (63.1)	101 (67.8)	142 (65.1)	60 (68.2)	0 (0.0)	202 (65.4)
	Strongly agree	23 (15.2)	3 (3.4)	3 (4.3)	25 (15.6)	4 (2.7)	23 (10.6)	5 (5.7)	1 (33.3)	29 (9.4)
	**Total**	**151 (100)**	**88 (100)**	**70 (100)**	**160 (100)**	**149 (100)**	**218 (100)**	**88 (100)**	**3 (100)**	**309 (100)**
**I would like to play a more active role in making decisions about my treatment for depression.**	Strongly disagree	0 (0.0)	0 (0.0)	0 (0.0)	0 (0.0)	0 (0.0)	0 (0.0)	0 (0.0)	0 (0.0)	0 (0.0)
	Disagree	2 (1.3)	3 (3.4)	0 (0.0)	1 (0.6)	4 (2.7)	4 (1.8)	1 (1.1)	0 (0.0)	5 (1.6)
	Somewhat disagree	1 (0.7)	9 (10.2)	1 (1.4)	1 (0.6)	10 (6.7)	9 (4.1)	2 (2.3)	0 (0.0)	11 (3.6)
	Somewhat agree	13 (8.6)	12 (13.6)	15 (21.4)	18 (11.3)	22 (14.8)	27 (142.)	13 (14.8)	0 (0.0)	40 (12.9)
	Agree	90 (59.6)	55 (62.5)	46 (65.7)	92 (57.5)	99 (66.4)	131 (60.1)	57 (64.8)	3 (100)	191 (61.8)
	Strongly agree	45 (29.8)	9 (10.2)	8 (11.4)	48 (30.0)	14 (9.4)	47 (21.6)	15 (17.0)	0 (0.0)	62 (20.1)
	**Total**	**151 (100)**	**88 (100)**	**70 (100)**	**160 (100)**	**149 (100)**	**218 (100)**	**88 (100)**	**3 (100)**	**309 (100)**
**I believe that using a short questionnaire to keep track of changes in my depression symptoms would help with my depression treatment**	Strongly disagree	0 (0.0)	0 (0.0)	0 (0.0)	0 (0.0)	0 (0.0)	0 (0.0)	0 (0.0)	0 (0.0)	0 (0.0)
	Disagree	5 (3.3)	5 (5.7)	2 (2.9)	6 (3.8)	6 (4.0)	9 (4.1)	3 (3.4)	0 (0.0)	12 (3.9)
	Somewhat disagree	5 (3.3)	10 (11.4)	6 (8.6)	9 (5.6)	12 (8.1)	17 (7.8)	3 (3.4)	1 (33.3)	21 (6.8)
	Somewhat agree	29 (19.2)	23 (26.1)	18 (25.7)	32 (20.0)	38 (26.5)	47 (21.6)	22 (25.0)	1 (33.3)	70 (22.7)
	Agree	95 (62.9)	45 (51.1)	39 (55.7)	92 (57.5)	87 (58.4)	125 (57.3)	53 (60.2)	1 (33.3)	179 (57.9)
	Strongly agree	17 (11.3)	5 (5.7)	5 (7.1)	21 (13.1)	6 (4.0)	20 (9.2)	7 (8.0)	0 (0.0)	27 (8.7)
	**Total**	**151 (100)**	**88 (100)**	**70 (100)**	**160 (100)**	**149 (100)**	**218 (100)**	**88 (100)**	**3 (100)**	**309 (100)**
**I believe that using a short questionnaire to keep track of changes in my depression symptoms would help me to talk to my doctor about my depression treatment**	Strongly disagree	0 (0.0)	0 (0.0)	0 (0.0)	0 (0.0)	0 (0.0)	0 (0.0)	0 (0.0)	0 (0.0)	0 (0.0)
	Disagree	1 (0.7)	4 (4.5)	0 (0.0)	1 (0.6)	4 (2.7)	4 (1.8)	1 (1.1)	0 (0.0)	5 (1.6)
	Somewhat disagree	4 (2.6)	11 (12.5)	3 (4.3)	4 (2.5)	14 (9.4)	13 (6.0)	5 (5.7)	0 (0.0)	18 (5.8)
	Somewhat agree	28 (18.5)	22 (25.0)	23 (32.9)	37 (23.1)	36 (24.2)	54 (24.8)	18 (20.5)	0 (0.0)	73 (23.6)
	Agree	99 (65..5)	48 (54.5)	41 (58.6)	96 (60.0)	92 (61.7)	127 (58.3)	59 (67.0)	1 (33.3)	188 (60.8)
	Strongly agree	19 (12.6)	3 (3.4)	3 (4.3)	22 (13.8)	3 (2.0)	20 (9.2)	5 (5.7)	2 (66.7)	25 (8.1)
	**Total**	**151 (100)**	**88 (100)**	**70 (100)**	**160 (100)**	**149 (100)**	**218 (100)**	**88 (100)**	**3 (100)**	**309 (100)**
**I believe that using a questionnaire as described in the scenario would help to understand my depression**	Strongly disagree	0 (0.0)	0 (0.0)	0 (0.0)	0 (0.0)	0 (0.0)	0 (0.0)	0 (0.0)	0 (0.0)	0 (0.0)
	Disagree	3 (2.0)	6 (6.8)	3 (4.3)	4 (2.5)	8 (5.4)	9 (4.1)	3 (3.4)	0 (0.0)	12 (3.9)
	Somewhat disagree	13 (8.6)	13 (14.8)	2 (2.9)	14 (8.8)	14 (9.4)	20 (9.2)	7 (8.0)	1 (33.3)	28 (9.1)
	Somewhat agree	23 (15.2)	18 (20.5)	22 (31.4)	28 (17.5)	35 (23.5)	43 (19.7)	19 (21.6)	1 (33.3)	63 (20.4)
	Agree	96 (63.6)	48 (54.5)	41 (58.6)	95 (59.4)	90 (60.4)	129 (59.2)	55 (62.5)	1 (33.3)	185 (59.9)
	Strongly agree	16 (10.6)	3 (3.4)	2 (2.9)	19 (11.9)	2 (1.3)	17 (7.8)	4 (4.5)	0 (0.0)	21 (6.8)
	**Total**	**151 (100)**	**88 (100)**	**70 (100)**	**160 (100)**	**149 (100)**	**218 (100)**	**88 (100)**	**3 (100)**	**309 (100)**

A majority of surveyed patients (65.1%) agree that they are willing to complete a questionnaire as described in the vignette. Some variations exist between health centers, with 15.9% of respondents from HKMC selecting somewhat disagree, compared with 3.3% from SMHC and 6.5% from FXMC. 15.2% of SMHC respondents strongly agree that they are willing, while 3.4% from HKMC and 4.8% from FXMC selected this response. Regarding the desire to play a more active role in making decisions about their treatment, agreement was strong across all MHCs, as shown in Table 9. When asked about the use of a questionnaire helping with depression treatment, again there was majority agreement across health centers. A majority of respondents across health centers also agreed that using a brief questionnaire could help them to discuss their depression with their clinician, though disagreement was somewhat higher at HKMC (Table 9). Finally, results related to the belief that using a brief questionnaire would help patients to understand their depression were consistent, with a majority agreeing with rates of disagreement again higher among HKMC respondents.

Results related to patient acceptability of eMBC based on a brief vignette are described in Table 10:

**Table 10:** Patient acceptability of eMBC

Survey	Response	Location			Age		Gender			Total: n (%)
		SMHC: n (%)	HKMC: n (%)	FXMC: n (%)	18–39 years	40+ years	F	M	Unknown	
**I would be willing to use Internet-based resources as described in the scenario to support me with managing depression**	Strongly disagree	0 (0.0)	1 (1.1)	0 (0.0)	0 (0.0)	1 (0.7)	0 (0.0)	1 (1.1)	0 (0.0)	1 (0.3)
	Disagree	4 (2.6)	10 (11.4)	7 (10)	3 (1.9)	18 (12.1)	14 (6.4)	6 (6.8)	1 (33.3)	21 (6.8)
	Somewhat disagree	13 (8.6)	15 (17.0)	9 (12.9)	12 (7.5)	25 (16.8)	26 (11.9)	11 (12.5)	0 (0.0)	37 (12.0)
	Somewhat agree	36 (23.8)	18 (20.5)	16 (22.9)	43 (26.9)	27 (18.1)	48 (22.0)	20 (22.7)	2 (66.7)	70 (22.7)
	Agree	82 (54.3)	40 (45.5)	36 (51.4)	85 (53.1)	73 (49.0)	115 (52.8)	43 (48.9)	0 (0.0)	158 (51.1)
	Strongly agree	16 (10.6)	4 (4.5)	2 (2.9)	17 (10.6)	5 (3.4)	15 (6.9)	7 (8.0)	0 (0.0)	22 (7.1)
	**Total**	**151 (100)**	**88 (100)**	**70 (100)**	**160 (100)**	**149 (100)**	**218 (100)**	**88 (100)**	**3 (100)**	**309 (100)**
**I would be willing to use a mobile app to track my symptoms if my doctor recommended it**	Strongly disagree	1 (0.7)	1 (1.1)	0 (0.0)	1 (0.6)	1 (0.7)	1 (0.5)	1 (1.1)	0 (0.0)	2 (0.6)
	Disagree	1 (0.7)	12 (13.6)	10 (14.3)	3 (1.9)	20 (13.4)	16 (7.3)	6 (6.8)	1 (33.3)	23 (7.4)
	Somewhat disagree	12 (7.9)	15 (17.0)	11 (15.7)	11 (6.9)	27 (18.1)	26 (11.9)	12 (13.6)	0 (0.0)	38 (12.3)
	Somewhat agree	27 (17.9)	16 (18.2)	12 (17.1)	29 (18.1)	26 (17.4)	35 (16.1)	19 (21.6)	1 (33.3)	55 (17.8)
	Agree	85 (56.3)	41 (46.6)	35 (50)	91 (56.9)	70 (47.0)	119 (54.6)	41 (46.6)	1 (33.3)	161 (52.1)
	Strongly agree	25 (16.6)	3 (3.4)	2 (2.9)	25 (15.6)	5 (3.4)	21 (9.6)	9 (10.2)	0 (0.0)	30 (9.7)
	**Total**	**151 (100)**	**88 (100)**	**70 (100)**	**160 (100)**	**149 (100)**	**218 (100)**	**88 (100)**	**3 (100)**	**309 (100)**
**I believe that I would be able to use an app to respond to a short questionnaire about my depression symptoms on a regular basis**	Strongly disagree	0 (0.0)	1 (1.1)	0 (0.0)	0 (0.0)	1 (0.7)	0 (0.0)	1 (1.1)	0 (0.0)	1 (0.3)
	Disagree	4 (2.6)	11 (12.5)	13 (18.6)	4 (2.5)	24 (16.1)	19 (8.7)	8 (9.1)	1 (33.3)	28 (9.1)
	Somewhat disagree	10 (6.6)	16 (18.2)	6 (8.6)	8 (5.0)	24 (16.1)	25 (11.5)	7 (8.0)	0 (0.0)	32 (10.4)
	Somewhat agree	22 (14.6)	16 (18.2)	14 (20.0)	29 (18.1)	23 (15.4)	33 (15.1)	18 (20.5)	1 (33.3)	52 (16.8)
	Agree	97 (64.2)	42 (47.7)	36 (51.4)	101 (63.1)	74 (49.7)	126 (57.8)	48 (54.4)	1 (33.3)	175 (56.6)
	Strongly agree	18 (11.9)	2 (2.3)	1 (1.4)	18 (11.3)	3 (2.0)	15 (6.9)	6 (6.8)	0 (0.0)	21 (6.8)
	**Total**	**151 (100)**	**88 (100)**	**70 (100)**	**160 (100)**	**149 (100)**	**218 (100)**	**88 (100)**	**3 (100)**	**309 (100)**

Regarding acceptability of eMBC among surveyed patients, a majority agree that they would be willing to use Internet-based resources to support managing their depression, willing to use an app to track their depression symptoms and in terms of their belief in their ability to use an app to respond to brief questionnaires regularly. Across all three categories, there is stronger agreement among SMHC respondents, while disagreement is higher among patients from HKMC and FXMC and higher disagreement among patients over 40 years (Table 10).

We provided focus group participants with the same vignette, eliciting responses related to perceived barriers and facilitators to using eMBC. A number of facilitators were identified. Broadly speaking, eMBC was seen as beneficial for both patients and clinicians:

*“The patient can understand and record himself by using the app, and the clinician can better understand the changes of the patient’s emotion. I think this win-win situation is pretty good.” (SMHC FG1)*

More specifically, the use of an app was seen as both economical in terms of saving time and money spent for in-person consultations, as described by a participant from SMHC:

*Especially during initial assessment, let’s say the app is universal[ly] used in any clinics, then it doesn’t matter where the patient will have the initial assessment, since the initial assessment is just some form tests. Patients can save the consultation fee and the time. That is the truth, I think using the app does good to me. (SMHC FG1)*

eMBC was also viewed as being more convenient:

*“it’s very convenient, in terms of the time. For example, if you can do it on your mobile phone, then you probably can finish it on the public transit (bus, subway), or do it on their way (to the hospital). Without the app, you need to do it in the hospital.”. (FXMC Young)*

Patients also acknowledged that eMBC might be more efficient for clinicians, helping to relieve some of the strain of a large number of clinical consultations each day. This was discussed by participants at SMHC:

*“The app does not just present the result by adding scores, but let’s say, it can reduce the time that clinician has to inquire patients, and also can direct the patients to adjust themselves, then it is beneficial. I think this is what the app should be like.” (SMHC FG1)*

And even have a positive impact on the reputation of the health center:

*“I think that the Mental Health Center in Hongkou will soon be famous and customers will have a degree of adhesion, they will come by themselves and the center’s reputation will increase”. (HK young group)*

Focus group participants also acknowledged that eMBC can help with understanding depression and self-management:

*“With this app, for example, you can have an assessment and test for yourself, then you can have some prevention, that is, you can mediate yourself, or go to the hospital for medical treatment, let’s say, an early planning”* (HKMC young group).

The collaborative nature of eMBC also appealed to patients:

*“The patient can learn more about himself and these quantitative indicators can show him what is happening now, or what the whole course of the depression will be. If combined (with) the clinician’s expertise to develop a treatment plan, will increase the patient’s confidence. I feel that the patient can know himself better in this way, since people may get nervous when they don’t know what is happening”. (HKMC Young group)*

Regarding whether they would adhere to eMBC instruction from their clinician, participant responses suggest that they value clinical advice:

*“Absolutely. Whatever the doctor said, I will adhere to. Listening to the clinician is very important, whatever the clinician said is right”. (SMHC FG2)*

Focus group participants also made suggestions about ways to improve the uptake and use of eMBC, including through promotion and awareness, as stated by a participants from FXMC: “*maybe there should be some promotion” (FX Young)*

*“There are so many apps in the market, how can I assess if an app is good or not?” (HK Young group)*

Regarding the need for training or education to support their use of eMBC, patient feedback was mixed:

One participant from SMHC stated that there is: *“No need (for training). People can understand that by themselves”. (SMHC FG2).*

While another stated: *“I think there needed to be some guidance from a professional clinician. The patients then can know how to use and when to use, so that they can know how to get the best results”. (SMHC FG2).*

Despite a number of facilitators identified by patients, not all focus group participant comments were positive about eMBC. Most prominently, participants voiced concerns about eMBC representing a replacement for in-person clinical contact, as illustrated by the following quotes from each MHC:

*“...that the app can shorten the time of consultation, is actually also a shortcoming. Each patient with depression is very distressed. He is not understood at home nor in the society. Hey, I am seeing the clinician because I want to talk more with the clinician from whom I can get support and understanding. Why do I have to shorten the interview time? I think this is very difficult to understand…To my opinion, I am seeing the clinician to discuss with him my symptoms, I can do the measure later if I need to.” (SMHC 01)*

*“I think this should be discussed with the doctors. After all if you use the app and learn by yourself based on your medical conditions, it’s not professional. In addition, the medications usually have side effects, and if there are changes caused by the side effects, I feel it’s better to discuss with the doctors face to face”. (FXMC Young)*

Consonant with some concerns voiced by clinicians, one patient from HKMC also spoke to concerns about eMBC eroding clinician expertise or authority:

*“If the clinician relies solely on the result of the app then give the patient a corresponding diagnosis, everyone can be a clinician, right?...I am worried that the app will cause the deterioration of the clinician’s professional medical level… (and they risk becoming) over-reliant on the app”. (HK Young group)*

Similarly, there is a perception that eMBC might lead to a loss of nuance in understanding or the human, interpretive element of a traditional clinical consultation. One SMHC participant said of using an app: *“Then there is no need for Dr. [Name] showing up. A robot can do that too.”*

Another stated:

*“The subjective things cannot be quantified. Not every patient with depression has the same symptom nor will they have the same emotion feelings.” (SMHC 01)*

Lack of conviction was also apparent in some focus group participants about clinical impact or accuracy of eMBC:

(Interviewer: Do you think using an app like this will improve your treatment and your mood?) “*I don’t think so. I think it’s just for reference”. (FX Young group)*

Another participant in the same focus group claimed: “*it’s better to communicate with the doctors in person, face to face”. (FX Young group).*

In a focus group among older adults at HKMC, participants also indicated their reluctance to use it, stating: *“I will not use it,” “I would still rather focus on my Buddhism”, and “I feel that it might not be that accurate.” (HKMC Senior group).*

Similarly to clinicians, some participants also questioned the ability of patients to accurately self-report symptoms, due to lack of insight or self-awareness, or even due a desire to see an improvement in scores:

*“I may not be able to fully express the changes I had in the past period. Because the feeling of depression is comparatively subtle, it is not easy to describe”. (SMHC FG2)*

*“...since the questions in the measure are fixed, patient might change his answer next time just because he wants to have a better score instead of indicating his real feelings.” (SMHC 01)*

From a more technical standpoint, concerns were also voiced about potential lack of long-term engagement with eMBC and about having to use or download a specific app:

*“I guess when the app comes out, at the beginning people would be like, I’ll download it to measure my moods, but you need to consider the follow-ups. Some people may just download it, use it for measurement, and then they’d be like oh there’s nothing wrong, so they may uninstall. This could happen” (FX Young group)*

## Discussion

This mixed methods situational analysis has identified several potential barriers and facilitators that will be essential for planning the implementation of MBC and eMBC at Shanghai mental health centers. These findings also make a substantial contribution to the literature on MBC implementation, which is growing in settings such as the United States [5, 7] and the United Kingdom [24] but remains limited in other contexts. Though the efficacy of standard MBC has been studied in China [8], factors influencing its implementation and that of eMBC have yet to be explored. The results of this situational analysis will directly inform the development of an eMBC implementation strategy to be tested via an RCT in Shanghai. The results also provide a broader contribution to the field of MBC implementation globally. Barriers and facilitators to MBC implementation exist at multiple levels and are heavily influenced by contextual factors. Understanding these factors can help to tailor multi-component implementation strategies to help promote successful implementation of MBC [5]. A number of barriers and facilitators of relevance to MBC and eMBC implementation at the system level and from the perspectives of clinicians and patients were identified in this analysis and are discussed below.

### Barriers

#### Organization and system level

Understanding organizational and system-level factors is essential for developing strategies to promote successful and lasting implementation [5]. We identified several potential barriers to MBC and eMBC implementation at the system level. Cost is a potential barrier that might affect the equitable delivery of MBC in Shanghai and, should MBC be scaled up, throughout China. The cost of medications varies greatly, and though many patients are insured and have access to subsidies when needed, the cost of some antidepressant medications may be prohibitive. This may in turn affect the full capacity of clinicians to adjust antidepressant medications based on MBC. Providers also cautioned that administration of outcome measures for standard MBC may involve an additional cost to patients. The cost of mental health treatment for patients in China can be substantial. A 2016 study [25] found that annual mental health costs more than tripled between 2005 and 2013, with direct out-of-pocket expenses for patients constituting approximately 40% of the total cost. The financial burden was higher among rural residents, with out-of-pocket mental healthcare expenditures accounting for approximately 50% of per capita disposable income. Costs associated with treating affective disorders were highest due to their elevated prevalence compared with other mental illnesses. The financial implications for patients will have to be considered when developing and adjusting treatment plans for patients based on MBC results in order to ensure equitable access to the highest quality evidence-based care.

Another potential barrier identified at the system level is the variation across centers in the likelihood of patients seeing the same clinician consistently. Lack of continuity of care may mean there is limited therapeutic alliance and impede consistent implementation of MBC. Continuity of care has been shown to have many health benefits [26] and may have an impact on implementation and outcomes related to MBC. This suggests that efforts should be made to ensure that patients are able to see the same clinician if they wish. In circumstances where this is unfeasible there is a need for systems that enable sharing of MBC and eMBC outcomes and treatment decisions among different clinicians.

There is also variation in EMR and Internet availability between centers. FXMC, the suburban center, uses paper records for outpatients and has less reliable Internet connection. The ability to integrate ‘measurement feedback systems’ such as those used in eMBC into existing EMR systems has been associated with improved use of MBC by clinicians in other contexts [5, 27]. The availability of EMR both within and across MHCs could therefore influence the implementation of MBC.

A final potential barrier at the system level is that currently outcome measures are only administered by psychometricians, meaning that clinicians may not be familiar with administering them and may not consider their administration to be within their purview. Being unfamiliar with both administering and interpreting outcome measures could represent a barrier to implementing MBC [24, 28], suggesting the need for enhanced training on the use of outcome measures for psychiatrists and other clinicians, in addition to awareness-raising about the availability and utility of these measures.

##### Provider perspective

While surveys showed a high level of endorsement for the value of using MBC, surveys and interviews showed that in current practice for both initial and follow-up assessment, clinicians primarily make diagnoses and treatment decisions based on patient or family report of symptoms and functional impairment and observation. Clinicians reported minimal existing use of standardized outcome measures. This is consistent with findings of previous studies from the United States [28], where most clinicians only used measures regularly if mandated by workplace policies.

Reports of being trained in MBC also varied considerably. Clinician report of low levels of training in MBC was identified as a barrier to application in the United Kingdom [24] and in the United States [29], underscoring the need for enhanced training and education about the use of measures.

Regarding clinician knowledge and beliefs about MBC, despite positive attitudes towards MBC by surveyed clinicians, in interviews a number of potential barriers were identified. Time, including a considerable number of hours per day spent in patient care and the perception that explaining and administering MBC will take time out of already busy schedules, emerged as a consistent concern. Specific to eMBC clinicians voiced concerns that using digital technologies such as apps might impinge on their personal time and raise patient expectations for them to be available outside of regular hours. Time and workload, unsurprisingly, have been identified as potential barriers to MBC implementation in other contexts [3, 5, 24, 28]. Strategies to mitigate this barrier include choosing measures that require a minimal burden for both patients and providers and selection of brief measures [5]. Using electronic technologies such as those employed in eMBC can also help to overcome time-related barriers [30].

A number of strategies are identified in the literature that might help to overcome some of these barriers related to negative attitudes or perceptions. Identification and involvement of local champions and opinion leaders, for example, is one approach to improve clinician attitudes towards MBC [5]. The involvement of senior clinicians at SMHCs, including via involvement in training sessions, may represent an important strategy for overcoming some negative attitudes towards MBC. Involvement of clinicians in developing MBC protocols was also identified as a strategy to overcome clinician reluctance to implement it [28].

Training followed by organizational support and supervision is also essential for improving clinician attitudes towards and use of MBC [3–5]. Policy recommendations on the implementation of MBC in the United States suggest that the inclusion of MBC in continuing medical education (CME) training would increase widespread adoption. This approach could also be taken in Shanghai and in China more broadly. As few training programs have been established and disseminated for MBC in mental health settings [3], there is an opportunity to develop and implement a training program that can be used widely among Chinese clinicians and adapted for delivery in other contexts. The APEC Digital Hub for Mental Health (mentalhealth.apec.org), which is led by several study investigators (RL, JM, EM, CN, AG), can serve as a platform for delivery and sharing of training programs for MBC that can be adapted and disseminated across the Asia Pacific region.

A potential barrier regarding the feasibility of MBC was the perception among clinicians that patients might have low capacity or efficacy to respond to measures. Clinicians suggested that patients might face time limitations or be reluctant to engage in MBC. Some also suggested that patients might not be able to accurately complete measures because they lack sufficient insight into their depression. Clinicians also noted that literacy challenges and the cognitive effects of severe depression might impede patient ability to complete measures. Concerns by clinicians that patients will have trouble understanding measures or will find them difficult or stressful have been identified as barriers elsewhere [28].

Though clinicians in this study found MBC to be generally acceptable, they were also wary that using MBC/eMBC would replace more traditional clinical judgement and practices, which they consider fundamental to psychiatric practice. Many suggested that MBC and eMBC could be used in a complementary way but should not replace standard clinical practice and decision making. Concerns about loss of clinical “formality” and the belief that MBC is inferior to more traditional clinical assessment has previously been identified as a barrier to MBC use by clinicians [5, 24]. In a study on clinician attitudes towards using standardized outcomes measures for child mental health, Garland et al. [28] found that about half of clinicians questioned the concept of objectively assessing change in treatment outcomes, arguing that it is reductive to try to interpret a range of human behaviour and experience using what they perceived as one paradigm.

Some Shanghai clinicians saw increased patient involvement in treatment planning and tracking depression outcomes as a threat to the status quo, which they worried might lead to patients questioning clinician expertise, disagreements about appropriate treatment decision-making, and reduction in trust in clinicians. Some also worried that patients “knowing too much”, for example about the potential side effects of medications, might make them less likely to adhere to recommended treatment.

##### Patient perspective

The perspective of patients is also essential to identifying barriers and drivers to MBC and eMBC implementation. Despite this, existing research about patient perspectives in MBC implementation, particularly in the field of mental health, is limited [5]. This situational analysis identifies several barriers and facilitators to MBC and eMBC implementation that are of relevance both in the Chinese context and more broadly.

Understanding patients’ current use of the Internet and smartphones can help to identify barriers related to patient uptake of eMBC. Overall a majority of patients reported high frequency of Internet use and Smartphone ownership. There is variation, however, between suburban and urban patients, with suburban patients reporting lower levels of use regardless of age. Patient reported familiarity with and use of apps generally and WeChat specifically varies between age categories, with 81.9% of patients under 40 years familiar with WeChat compared with 49% over 40 years. The large proportion of Internet users in China has led to an increasing interest in use of digital health technologies for depression care [31]. Though a majority of patients have used Internet-based resources to access health and mental health information, lower use of the Internet for health information was reported by patients at the suburban center (FXMC). High levels of Internet, smartphone and app use are promising for eMBC implementation success. However, the variation in usage among patients in suburban areas should be considered in implementation planning and may suggest that, in terms of broader scale-up, Internet, smartphone and app usage might vary in rural areas of China and among patients of different age groups.

Though general acceptability of eMBC by patients was high, some were concerned that eMBC would replace in-person contact with clinicians, which was highly valued. The therapeutic alliance with their clinician and the opportunity to discuss their depression symptoms openly, which may not be possible in other aspects of their lives, was seen as important. Similar to concerns expressed by clinicians, some patients were also worried that eMBC might undermine or replace clinician expertise, believing that eMBC could work as a complementary approach but should not be used to replace formal clinical expertise or the individualized, personal side of human interaction. The perception of patients that eMBC might actually take away from the therapeutic alliance and patient-clinician interaction could be addressed by awareness-raising efforts to inform patients that MBC approaches are in fact effective for increasing shared decision making in depression care and enhancing communication between patients and providers [2, 3, 32].

Similarly to clinicians, some patients expressed concerns about the capacity of patients with depression to accurately respond to measures when symptoms might impact their cognition or insight. Patient symptoms, in addition to barriers created by lack of accessible delivery of outcome measures (e.g. for patients who are visually impaired) may act as barriers to equitable engagement of patients in MBC and eMBC [5]. Accessibility considerations as well as options such as patient navigators or liaisons can help to minimize these challenges [5]. Given the important role of patient family members in supporting patients during consultations, family members could be engaged to support patients to complete outcome measures when needed.

Finally, some patients also voiced skepticism about the potential for long-term engagement with eMBC. Sustained use of mental health apps, even with high volumes of downloads, has been identified as a challenge in the field of digital mental health [33, 34]. In the case of eMBC, the integration of the use of electronic outcome measures into regular appointments with clinicians might help patients to sustain their engagement over their treatment course. Further, the integration of eMBC with a supported self-management programs, as is planned in this study, might further help to sustain the engagement of patients in tracking their depression outcomes for the duration of the self-management intervention.

### Facilitators

#### Organization and system level

A key facilitator for the implementation of eMBC in Shanghai mental health centers is that, despite potential challenges in centers such as FXMC, the use of EMR and availability of computers and Internet at MHCs is largely widespread. Though it is certainly possible to implement standard MBC using paper and pencil, it is preferable to integrate outcome measure results into EMR [2, 5]. This can also facilitate collaboration and continuity across clinicians when patients see multiple providers [2].

##### Provider perspective

In a qualitative study about clinician attitudes towards MBC in the United States, attitudes related to the clinical utility of MBC were varied, with approximately half stating that they believed using MBC was best practice while others were opposed to its use [28]. Clinicians in Shanghai seem similarly divided. Despite the barriers associated with knowledge and beliefs described above, surveyed providers showed positive attitudes regarding the validity, reliability and effectiveness of MBC. Many interviewed clinicians also displayed positive attitudes and beliefs about MBC, describing it as more standardized, comprehensive and systematic than standard care. These positive attitudes are promising for the uptake and adoption of MBC by Shanghai clinicians. Evidence also suggests that using MBC and reviewing aggregate MBC data over time can be used by individual clinicians for professional development [2, 3]. Therefore, use of MBC and first-hand experience of its clinical utility may help to further improve clinician attitudes at individual, organizational and systems levels.

Many clinicians also expressed the belief that the increased engagement of patients via MBC could empower patients, improve their awareness and insight into their depression and treatment trajectory, and ultimately improve treatment adherence and outcomes. Patient empowerment has been identified as a benefit of MBC [2]. Again, there is some disparity among clinicians about their perception of patient empowerment as a benefit compared with concerns about the implications of increased patient awareness and autonomy. These discrepancies will have to be navigated in planning for MBC implementation and addressed via training and supervision.

Regarding the feasibility of MBC and eMBC implementation, clinicians identified several factors that they believe would help to facilitate the use of MBC in their own practice, including proper training and resources, a dedicated staff person responsible for facilitating MBC, taking a team-based approach, and the availability of private space to complete measures. These factors are identified as facilitators to MBC implementation in other contexts [5, 29].

Importantly, before being asked about eMBC specifically, clinicians identified automation or electronic delivery as facilitators to implementing MBC, helping to overcome perceived barriers of implementing standard MBC including time constraints. When specifically asked about eMBC they believed that it might have benefits including improved patient knowledge about depression, improved treatment adherence and reduction in self-stigma. This suggests that eMBC might be more acceptable and feasible for clinicians compared with standard MBC. Clinicians suggested that marketing or awareness-raising programs to support the introduction of eMBC combined with adequate training for both clinicians and patients would help to facilitate its uptake and implementation. Adequate training and supervision along with workplace policies are identified as facilitators of MBC implementation [4, 5].

##### Patient perspective

Overall, patients participating in this study demonstrated strong overall willingness to use outcomes measures and believed that MBC can help them manage symptoms and help them become more active in their treatment. They perceived eMBC to be efficient and convenient with the potential to improve their understanding of depression and their self-management capacity. Patients also agreed with clinicians that marketing or promotion and awareness programs about eMBC should be implemented in order to facilitate patient awareness and uptake of MBC and eMBC. There is little existing literature about facilitating factors for patient uptake and engagement in MBC. This study will therefore help to strengthen the evidence-based on patient-level factors influencing MBC and eMBC implementation.

### Limitations

Through this situational analysis we sought to develop a comprehensive understanding of the context of implementation for MBC and eMBC for depression in Shanghai mental health centers. A potential limitation is that the current study does not extensively describe systemic factors that might influence long-term implementation and scale-up of MBC. Though a comprehensive evaluation of broad mental health system factors is outside the scope of the current study, we have collected data in this area that we intend to publish separately.

Another limitation is the low sample size for some patient focus groups, which resulted from challenges with recruitment at some health centers. Though we anticipated higher levels of participation in patient focus groups, the mixed methods nature of this study helps to ensure that patient perspectives are well-represented. The value of mixed methods research is to allow for data triangulation, and we feel that the survey results, in addition to the focus group data, adequately captures the patient perspective.

Finally, this situational analysis examines contextual factors across three mental health centers in a city with a population of over 24 million people and may therefore not be representative of the population of Shanghai. We have included diverse health centers- one large tertiary hospital, one urban health center and one suburban health center- to help to ensure that this analysis captures the broad context of mental health care delivery in Shanghai. Further research, however, may be needed to understand contextual factors influencing scale-up of MBC to regions outside of Shanghai.

## Conclusions

This situational analysis provides a comprehensive understanding of the potential barriers and facilitators to MBC and eMBC implementation in mental health centers in Shanghai, China at the organization and system level and from the perspective of clinicians and patients. This study makes an important contribution to field of MBC and implementation of evidence-based practices for depression. Though research about implementing MBC is growing in contexts such as the United States and United Kingdom, to our knowledge this is the first study to assess MBC implementation factors in China or in Asia more broadly. This study also specifically addressed factors influencing the implementation of eMBC, which is under-explored. The findings of this analysis will be used to develop and test, via an RCT, an implementation strategy for eMBC in Shanghai mental health centers. The results will help to inform scale-up throughout China and may help to inform implementation of MBC and eMBC in other global settings. The training program for clinicians developed through this study can be adapted to other settings and will be disseminated to other Asia Pacific contexts via the APEC Digital Hub for Mental Health.

Essential to the successful implementation of eMBC is ensuring that patients have equitable access to the benefits of evidence-based care for depression. Several factors that might influence equity were identified in this study. The cost of antidepressant medications and of the administration of outcome measures may make MBC and related treatment recommendations inaccessible to some patients. The use of and access to digital technology that is key to eMBC implementation might be disparate across locations and age groups, meaning that patients in more rural areas or in older demographics might be excluded. Accessibility of eMBC might also act as a barrier to equitable access, with general and digital literacy and disabilities such as visual impairment acting as barriers. These factors, while not always easy to address via one intervention, should be considered when planning for MBC and eMBC implementation and is planning for scale-up beyond the Shanghai context.

## Data Availability

The datasets used and/or analysed during the current study are available from the corresponding author on reasonable request.

## Abbreviations

EBP: Evidence-based practice
eMBC: Enhanced Measurement based care
FXMC: Fengxian Mental Health Centre
GMH: Global mental health
HKMC: Hongkou Mental Health Centre
MBC: Measurement based care
MDD: Major depressive disorder
RCT: Randomized controlled trial
SA: Situational analysis
SHMC: Shanghai Mental Health Centre
